# Calcium cytotoxicity sensitizes prostate cancer cells to standard-of-care treatments for locally advanced tumors

**DOI:** 10.1038/s41419-020-03256-5

**Published:** 2020-12-07

**Authors:** Alessandro Alaimo, Marco Lorenzoni, Paolo Ambrosino, Arianna Bertossi, Alessandra Bisio, Alice Macchia, Eugenio Zoni, Sacha Genovesi, Francesco Cambuli, Veronica Foletto, Dario De Felice, Maria Virginia Soldovieri, Ilaria Mosca, Francesco Gandolfi, Matteo Brunelli, Gianluca Petris, Anna Cereseto, Alvaro Villarroel, George Thalmann, Francesco Giuseppe Carbone, Marianna Kruithof-de Julio, Mattia Barbareschi, Alessandro Romanel, Maurizio Taglialatela, Andrea Lunardi

**Affiliations:** 1 Department of Cellular, Computational and Integrative Biology (CIBIO), University of Trento, Trento, Italy; 2 Department of Science and Technology (DST), University of Sannio, Benevento, Italy; 3 Department for BioMedical Research, Urology Research Laboratory, University of Bern, Bern, Switzerland; 4 Department of Medicine and Health Sciences, University of Molise, Campobasso, Italy; 5 Department of Pathology AOUI, University of Verona, Verona, Italy; 6 Biofisika Institute (CSIC, UPV/EHU), University of the Basque Country, Leioa, Spain; 7 Department of Urology, Inselspital, Bern University Hospital, University of Bern, Bern, Switzerland; 8 Unit of Surgical Pathology, Santa Chiara Hospital, Trento, Italy; 9 Department of Neuroscience, University of Naples “Federico II”, Naples, Italy

**Keywords:** Targeted therapies, Prostate cancer

## Abstract

Therapy resistance is a major roadblock in oncology. Exacerbation of molecular dysfunctions typical of cancer cells have proven effective in twisting oncogenic mechanisms to lethal conditions, thus offering new therapeutic avenues for cancer treatment. Here, we demonstrate that selective agonists of Transient Receptor Potential cation channel subfamily M member 8 (TRPM8), a cation channel characteristic of the prostate epithelium frequently overexpressed in advanced stage III/IV prostate cancers (PCa), sensitize therapy refractory models of PCa to radio, chemo or hormonal treatment. Overall, our study demonstrates that pharmacological-induced Ca^2+^ cytotoxicity is an actionable strategy to sensitize cancer cells to standard therapies.

## Introduction

Prostate cancer (PCa) represents the second most common type of cancer and the fifth leading cause of death in men in the industrialized countries^1^. Defeating metastatic PCa (mPCa) is considered a primary target to override tumor lethality^2^, however, the identification of novel therapies and the development of more effective clinical protocols for the treatment of locally advanced/high-risk tumors would significantly contribute to the reduction of PCa mortality, since these tumors frequently progress to the incurable stage of the disease^3^.

Intracellular calcium overload is one of the most powerful mechanisms of cell death in both normal and malignant cells^4^. Although several molecular mechanisms protect PCa cells by Ca^2+^ cytotoxicity^5–9^, different Ca^2+^ permeable channels result overexpressed in PCa. The Transient Receptor Potential cation channel subfamily M member 8 (TRPM8) is particularly interesting in the setting of PCa. Three times more selective for Ca^2+^ than K^+^ and Na^+^^10,11^, TRPM8 expression increases in primary prostate cancer compared to the benign counterpart^12–15^, while it is almost invariably lost in metastatic CRPC (mCRPC)^14^. Overexpression of TRPM8 in prostate tumor cells determines an enrichment of operating channel at the plasma membrane^15^, which has been shown to be functional for generating oncogenic stimuli associated with increased Ca^2+^ signaling^16–20^.

Since Ca^2+^ signaling can promote tumor cell death or survival depending by its nature, pharmacologic interventions directed against specific Ca^2+^ permeable channels abnormally expressed in cancer cells^21–23^ can represent a valid alternative to induce tumor cytotoxicity by containing the side effects at acceptable levels.

Here, by applying a multidisciplinary approach to a tailored in vitro/ex vivo preclinical platform, we demonstrate that pharmacological activation of TRPM8 in primary and hormone naïve metastatic models of PCa sensitizes prostate cancer cells to standard-of-care clinical protocols for the treatment of locally advanced PCa, thus pointing out the relevance of ion channels as therapeutic targets in oncology.

## Results

### TRPM8 is highly expressed in stage III/IV prostate cancers

**Fig. 1:**
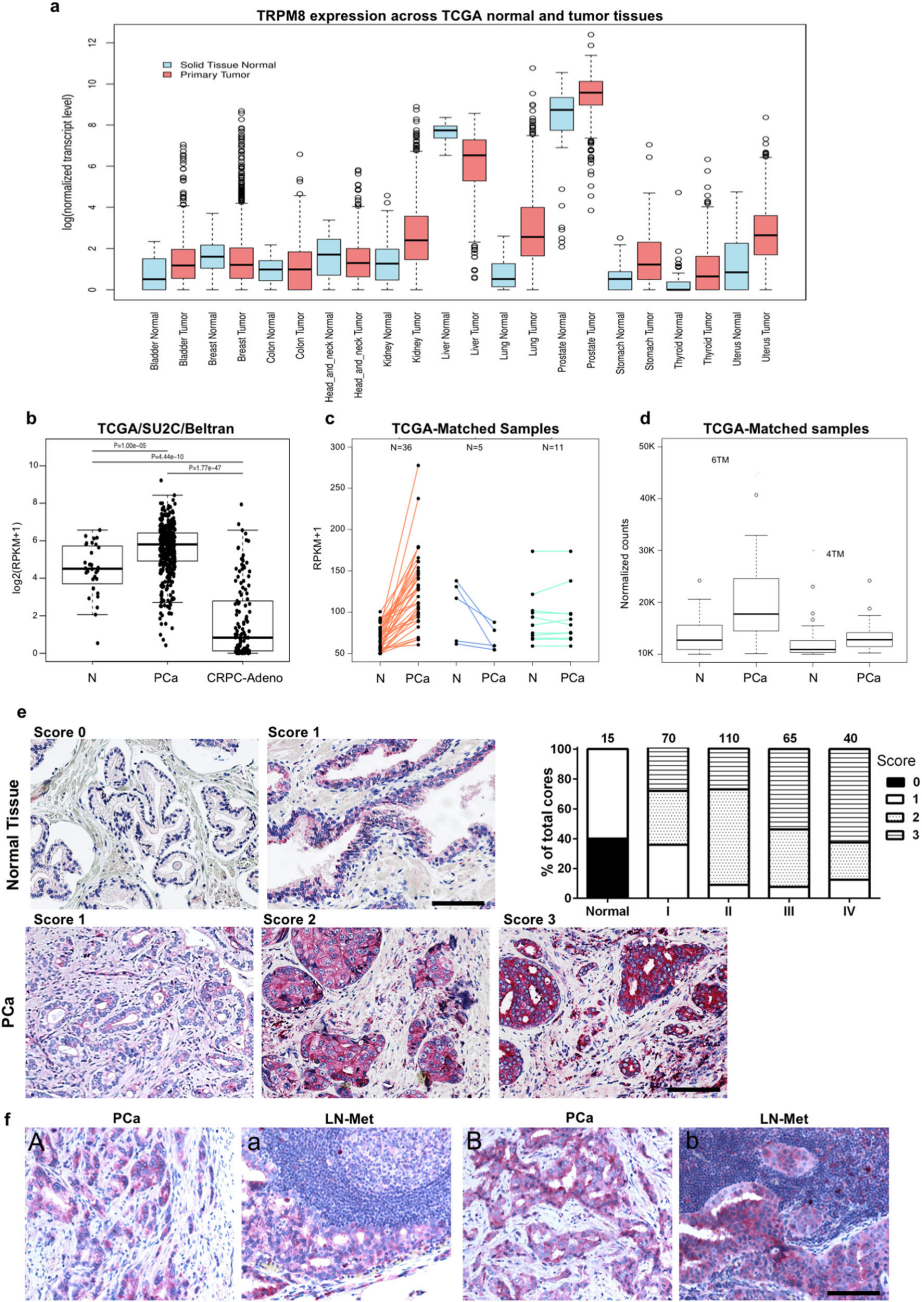
TRPM8 expression in human PCa. **a** TCGA RNA-seq dataset showing TRPM8 expression levels in normal tissues and related primary tumors. **b** TCGA, SU2C, and Beltran RNA-seq datasets analysis stating TRPM8 expression levels in benign prostate tissue, primary PCa and castration resistant metastatic adeno-PCa. Data were analyzed using a two-tailed Wilcoxon–Mann–Whitney test with a significance level set at 5%. **c** TRPM8 mRNA levels in 52 matched normal and adjacent PCa samples showing increased expression of TRPM8 in PCa compared to adjacent normal tissue in 36 cases, reduction in 5 and comparable levels in 11. **d** Relative amount of PM-associated 6TM (full length) and ER-associated 4TM TRPM8 transcript isoforms in 52 matched normal (N) and primary PCa (PCa) samples, as retrieved in TCGA RNA-seq dataset. **e** TRPM8 immunostaining score of a commercially available PCa tissue microarray (TMA). TRPM8 immunostaining was scored as weak (0), moderate (1), high (2), or very high (3) on 5 normal prostate cores and 171 PCa cores representing 57 different cases (3 cores × tumor). Representative images of scored normal prostate tissue and prostate adenocarcinoma cores are shown. Results are presented as percentage of tumors scored 0-to-3 respect to tumor stage. Stage I: score 1 = 36%; score 2 = 36%; score 3 = 28%; stage II: score 1 = 9%, score 2 = 64%, score 3 = 27%; stage III: score 1 = 8%; score 2 = 38%; score 3 54%; stage IV: score 1 = 12%, score 2 = 25%, score 3 = 63%. Scale bars, 100 μm. **f** TRPM8 immunostaining of matched primary PCa (A, B) and hormone naïve lymph node metastases (a, b). Scale bars, 100 μm.

Taking advantage of The Cancer Genome Atlas (TCGA) repository, we have compared TRPM8 expression in a panel of normal epithelial tissues and derived primary tumors (Fig. 1a). Among normal epithelia, TRPM8 expression in the prostate exceeds the expression of the channel in all the tissues analyzed (Fig. 1a). With the exclusion of the liver where the amount declines upon malignant transformation, TRPM8 mRNA rises in several types of tumors with groups of bladder, breast, kidney and lung cancer samples characterized by particularly high expression of the channel (Fig. 1a). Prostate tumors show in absolute the highest levels of TRPM8 (Fig. 1a).

To accurately profile TRPM8 expression in normal and tumor prostate tissue, we have interrogated widely used RNA-seq^24–26^ and microarray PCa datasets^27,28^. All datasets depict a high level of heterogeneity of *TRPM8* expression between tumors, nevertheless, invariably, the amount of the transcript rises in primary tumor samples compared to benign prostate tissues, to drastically fall in castration resistant metastatic PCa (Fig. 1a, b and Supplementary Fig. S1a, b).

Read mapping demonstrates that two TRPM8 mRNA isoforms (UCSC knownGene table GRCh37/hg19) are expressed in human prostate specimens, encoding, respectively, the full-length plasma membrane (PM) channel (6TM TRPM8) and the endoplasmic reticulum (ER) associated shorter form of the protein (4TM TRPM8) (Supplementary Fig. S1c, d). Analysis of 52 paired normal and tumor prostate samples annotated in the TCGA dataset, formally demonstrates: (i) the increased expression of *TRPM8* in the vast majority (36 out of 52) of primary PCa compared to adjacent benign prostate tissue (Fig. 1c), and (ii) the prevalent expression of the full-length 6TM TRPM8 isoform in PCa (Fig. 1d).

Finally, analysis of *TRPM8* expression in PCa samples grouped according to the Gleason score reveals no significant correlation between transcript amount and aggressiveness of primary tumors (Supplementary Fig. S1e). By contrast, elevated *TRPM8* expression associates with an improved overall survival (OS) of PCa patients (Supplementary Fig. S1f).

To refine our knowledge about TRPM8 expression in PCa, histological prostate specimens have been analyzed by immunohistochemistry. A commercially available PCa TMA (US Biomax Inc. PR208a) has been stained with the Alomone antibody ACC-049 (Fig. 1e, Supplementary Fig. S2a and Supplementary Fig. S3b, c, e). TRPM8 immunohistochemistry specifically marks the epithelial compartment of the prostate tissue (Fig. 1e, upper panels), with cancer cells (HMWCKs negative lumens) more intensely stained than the adjacent normal epithelium (HMWCKs positive lumens) (Supplementary Fig. S2b). TMA semi-quantification through pathologist visual analysis (score 0 = weak, 1 = moderate, 2 = high, and 3 = very high) confirms the heterogeneity of TRPM8 amount among tumors, with score 2–3 more frequently associated with advanced stages of the disease (Fig. 1e and Supplementary Fig. S2c, e).

**Fig. 2:**
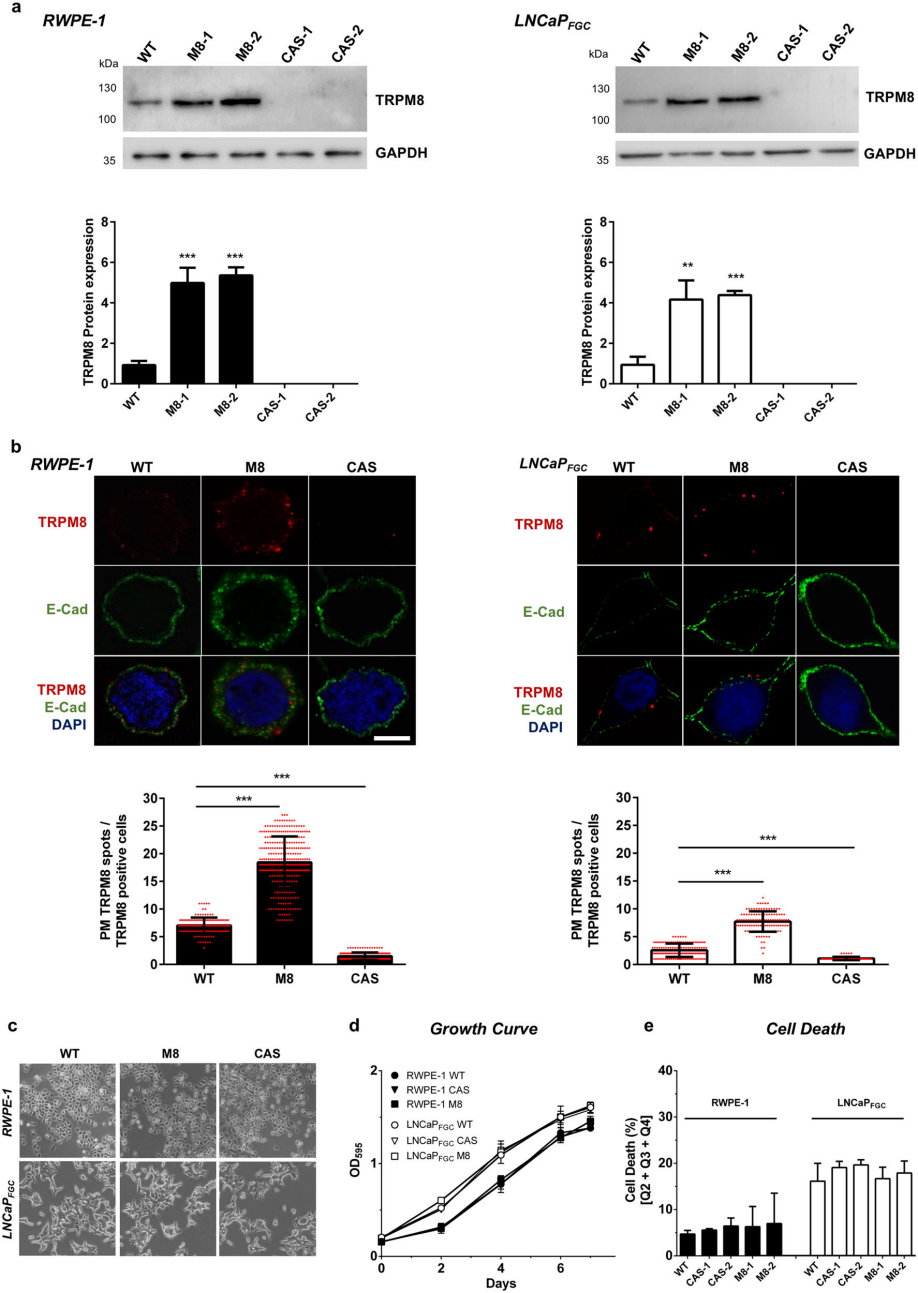
Modeling different levels of TRPM8 in RWPE-1 and LNCaP_FGC_ prostate cell lines. **a** Western blot analysis and quantification of full-length 6TM TRPM8 amount in RWPE-1 and LNCaP_FGC_ cell lines expressing endogenous (WT), overexpressed (M8) or knocked-out (CAS) levels of the protein. **b** Immunofluorescence analysis and quantification of PM-associated full-length 6TM TRPM8 in RWPE-1 and LNCaP_FGC_ cell lines with endogenous (WT), overexpressed (M8), or knocked-out (CAS) levels of the channel. For quantification of PM TRPM8 positive cells a total of 6000 cells were counted from different fields. Scale bar, 5 μm. **c–e** Morphology (**c**), growth (**d**), and cell death (**e**) analyses of RWPE-1 and LNCaP_FGC_ cell lines with endogenous (WT), overexpressed (M8), or knocked-out (CAS) levels of TRPM8. Error bars, mean ± SD. Experiments were performed in triplicate; data were analyzed using a two-tailed Student’s *t*-test. **P ≤ 0.01; ***P ≤ 0.001.

Lastly, parallel TRPM8 immunostaining in primary prostate tumors and hormone naïve lymph node metastases collected from the same patient shows comparable amount of the channel (Fig. 1f and Supplementary Fig. S2d).

Overall, our findings demonstrate that: (i) full-length plasma membrane 6TM TRPM8 is the most expressed isoform of the channel in PCa; (ii) TRPM8 immunostaining scores high in a relevant percentage of stage III/IV PCa; and (iii) hormone naïve local lymph node metastases express similar levels of TRPM8 compared to paired primary tumors.

### Modeling TRPM8 level heterogeneity to study prostate cells response to channel gating

In order to establish a preclinical in vitro platform where studying the impact of TRPM8 targeting on normal and malignant prostate cells expressing different amount of the channel, we profiled TRPM8 expression in a panel of commonly used immortalized and metastatic human prostate cell lines. Endpoint PCR studies with isoform-specific sets of primers (Supplementary Fig. S1d) define 6TM TRPM8 as the more common TRPM8 transcript in both immortalized (RWPE-1 and PWR-1E) and metastatic PCa cell lines (VCaP, LNCaP, LNCaP_FastGrowingClone_, MDA-PCa-2b, C4-2, PC3, DU-145, and NCI-H660), while the shorter 4TM-coding mRNA variant is detectable only in the LNCaP_FGC_ cells (Supplementary Fig. S3a, d). Of note, 6TM TRPM8 is mainly expressed in androgen sensitive immortalized and metastatic human prostate cell lines (RWPE-1, VCaP, LNCaP, LNCaP_FGC_, MDA-PCa-2b, C4-2) (Supplementary Fig. S3a, d). Western blotting analysis with two antibodies against TRPM8 confirms the mRNA expression analyses (Supplementary Fig. S3b, c, e) and shows an unexpectedly abundant 6TM TRPM8 protein amount in immortalized (RWPE-1 and PWR-1E) prostate cells irrespective of the low amount of the transcript (Supplementary Fig. S3a, b). In line with these data, analysis of benign, primitive PCa and mCRPC samples profiled in the TCGA dataset demonstrates a significant correlation between TRPM8 expression and androgen receptor (AR) transcriptional score (Supplementary Fig. S3f, upper panels), with the highest level of statistical significance observed between TRPM8 mRNA levels and expression of primary AR targeted genes such as *NKX3.1* and *KLK2* (Supplementary Fig. S3f, middle and lower panels).

As first step in the generation of an in vitro platform where testing the impact of TRPM8 pharmacology on first-line clinical protocols adopted for the treatment of locally advanced/high-risk PCa, immortalized androgen sensitive RWPE-1 (Supplementary Fig. S4) and hormone naïve lymph node metastatic LNCaP_FGC_ cell lines have been genetically engineered to model different levels of TRPM8 expression. In details, full-length (3.312 bp) cDNA encoding the 6TM TRPM8 isoform (hereinafter TRPM8) has been cloned from both immortalized RWPE-1 and metastatic LNCaP_FGC_ cells, controlled by sequencing, and stably integrated through viral transduction in the genome of RWPE-1 and LNCaP_FGC_ cells, thus generating respectively the RWPE-1 M8 and LNCaP_FGC_ M8 sublines. TRPM8 overexpression leads to 4-to-6 times more protein, and almost three times more TRPM8 loading at the plasma membrane, in RWPE-1 M8 and LNCaP_FGC_ M8 cells compared to RWPE-1 and LNCaP_FGC_ (Fig. 2a, b). Of note, RWPE-1 cells show a greater amount of TRPM8 at the plasma membrane compared to the LNCaP_FGC_ cells (Fig. 2b). Furthermore, TRPM8 has been knocked-out in both RWPE-1 and LNCaP_FGC_ lines through CRISPR-Cas9 methodology to generate the TRPM8-null RWPE-1 (RWPE-1 CAS) and LNCaP_FGC_ (LNCaP_FGC_ CAS) sublines (Fig. 2a, b).

No significant differences in cell morphology (Fig. 2c), proliferation (Fig. 2d), and survival (Fig. 2e) have been observed by comparing RWPE-1 and LNCaP_FGC_ cell lines expressing endogenous, overexpressed or knocked-out levels of TRPM8.

**Fig. 3:**
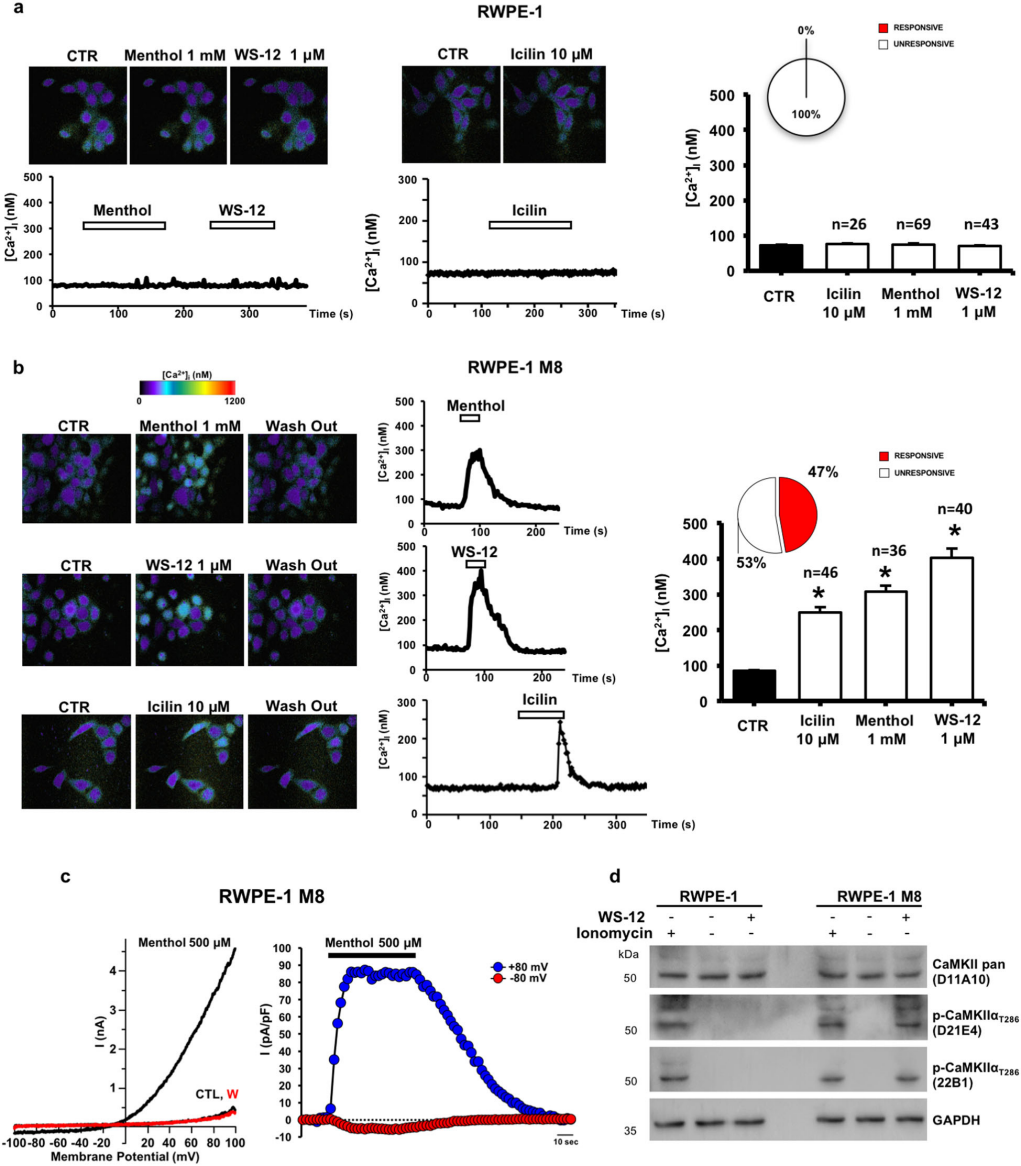
TRPM8 channel activity in RWPE-1 prostate cells. **a** Representative images (upper panels) and traces (lower panels) showing [Ca^2+^]_i_ changes under control solution (CTR) or upon perfusion with menthol (1 mM), WS-12 (1 μM), or icilin (10 μM) on RWPE-1 cells. Time of drugs exposure is indicated by the bar on top of the traces. Quantification of [Ca^2+^]_i_ peaks measured upon perfusion with TRPM8 activators is reported on the right panel (*n* = number of analyzed cells). The inset graph indicates the quantification of the total % of cells responsive to tested drugs. **b** Representative images (left panels) and traces (middle panels) showing [Ca^2+^]_i_ under control solution (CTR), upon perfusion with menthol (1 mM), WS-12 (1 μM), or icilin (10 μM), or after drugs washout on RWPE-1 M8 cells. Time of drugs exposure is indicated by the bar on top of the traces. Quantification of the [Ca^2+^]_i_ peaks measured upon perfusion with TRPM8 activators is reported on the right panel (*n* = number of analyzed cells). The inset graph indicates the percentage of cells responsive to the drugs tested. **c** Left panel, representative traces of currents evoked by a 100 ms voltage ramp ranging from −100 mV to +100 mV applied every 4 s in control solution (CTL), during application of menthol (500 μM) or after drug washout (W). Right panel, representative time-courses of currents recorded at +80 mV (blue symbols) or −80 mV (red symbols) in single RWPE-1 M8 cells upon exposure to menthol (500 μM). Time of menthol exposure is indicated by the line on top of the traces. **d** Western blotting analysis with two independent antibodies (D21E4 and 22B1) showing CaMKIIα activation (phosphorylation of Thr286) following WS-12 treatment of RWPE-1 M8 cells. Cells treated with ionomycin were used as positive control for calcium dependent CaMKIIα phosphorylation. Error bars, mean ± SEM. Experiments were performed in at least three experimental sessions; data were analyzed using a two-tailed Student’s *t*-test. *P ≤ 0.05.

TRPM8 is a cation channel whose function at the plasma membrane is primarily associated with intracellular Ca^2+^ influx. In order to assess TRPM8 activity in the parental and newly generated RWPE-1 cell lines, we have measured the changes in intracellular Ca^2+^ concentrations upon administration of different TRPM8 agonists. Well-studied TRPM8 activators such as menthol (1 mM) and icilin (10 μM), or the most potent WS-12 (1 μM)^29,30^, failed to trigger significant increase in intracellular calcium levels ([Ca^2+^]_i_) in RWPE-1 cells (Fig. 3a). A rapid increase in [Ca^2+^]_i_ was instead measured in RWPE-1 M8 cells ([Ca^2+^]_i_ was 84.9 ± 2.8 nM at basal levels and 307.3 ± 17.2, 402.5 ± 27.4, or 249.0 ± 15.2 nM after menthol, WS-12 or icilin exposure, respectively; *n* = 122; *p* < 0.05 between basal or drug-evoked [Ca^2+^]_i_ levels) (Fig. 3b). Drug-evoked [Ca^2+^]_i_ increase is fully reversible upon drugs washout (Fig. 3b). To prove that the recorded [Ca^2+^]_i_ changes effectively depended on TRPM8 channel activation, electrophysiological recordings have been performed in these cells upon menthol application. As reported in Fig. 3c, in RWPE-1 M8 cells menthol triggered the activation of robust currents showing an outwardly rectifying profile characteristic for TRPM8 activity (current densities at +80 mV were 83.5 ± 3.7 pA/pF; *n* = 4), an effect fully reversible upon drug washout (Fig. 3c). Phosphorylation of calcium-calmodulin dependent protein kinase IIα (CAMKIIα) on Threonine 286 demonstrates the induction of Ca^2+^ signaling in RWPE-1 M8 following TRPM8 activation with WS-12 (Fig. 3d).

**Fig. 4:**
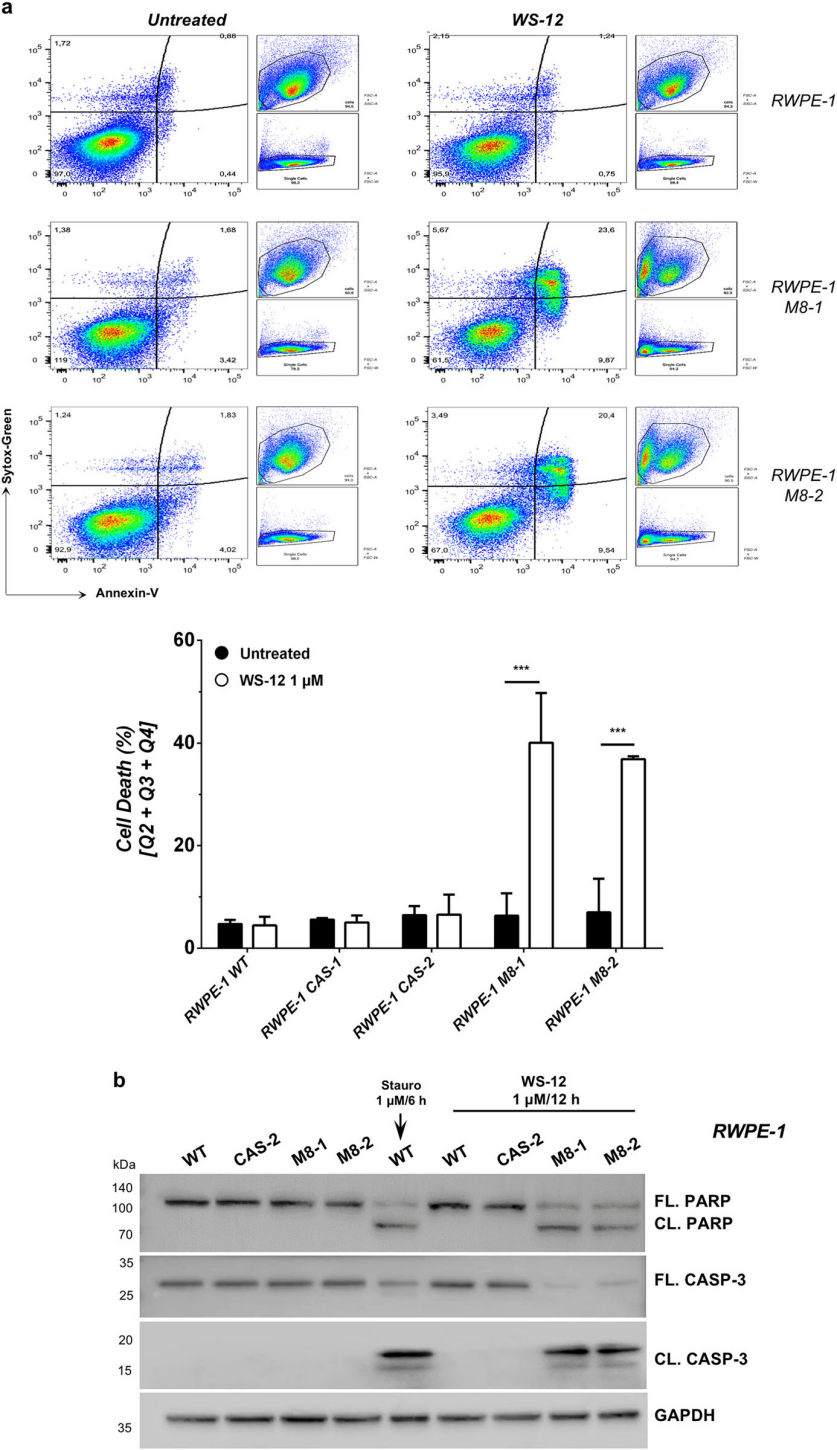
RWPE-1 response to TRPM8 agonist WS-12. **a** Cell death response by FACS (Annexin-V; Sytox-Green) in RWPE-1 cells expressing endogenous, increased (M8) or knocked-out (CAS) TRPM8 levels following 12 h WS-12 (1 μM) administration. Quantification is reported as percentage of total cells (lower panel). **b** Western blotting analysis showing molecular signature of apoptotic cell death (Caspase-3 and PARP cleavage). Staurosporine was used as positive control. Error bars, mean ± SD. Experiments were performed in triplicate; data were analyzed using a two-tailed Student’s *t*-test. ***P ≤ 0.001.

Excessive Ca^2+^ influx from the extracellular space and uncontrolled Ca^2+^ release from the intracellular storages are well-established potent inducers of apoptotic cell death^4^. To study the biological outcome of TRPM8 activation in nontumor prostate cells, RWPE-1 cell lines expressing endogenous, increased (M8), or knocked-out (CAS) levels of the channel have been treated or not with the most potent TRPM8 agonist WS-12 and analyzed by Fluorescence Activated Cell Sorting (FACS) for the apoptotic marker Annexin-V. Results demonstrate that 12 h of 1 μM WS-12 treatment are sufficient to trigger a robust apoptotic program in almost 40% of RWPE-1 M8 cells, while cell death rate does not change upon treatment in RWPE-1 cells expressing either endogenous or knocked-out (CAS) levels of the channel (Fig. 4a and Supplementary Fig. S5a). Menthol or icilin administration produces similar responses, even though the percentage of apoptotic RWPE-1 M8 cells is consistently less compared to WS-12 (Supplementary Fig. S5b). Western blotting analysis for the apoptotic molecular markers Caspase-3 and PARP fully confirms the induction of a potent apoptotic cell death program induced by WS-12 in RWPE-1 M8 prostate cells (Fig. 4b).

Overall, these data establish that increased amount of TRPM8 at the plasma membrane of prostate cells can produce intense intracellular Ca^2+^ currents and the consequent activation of a programmed cell death program upon administration of potent TRPM8 agonists.

### Ca^2+^ cytotoxicity improves therapy efficacy in in vitro/ex vivo models of PCa

Lack of human PCa cell lines derived from primary tumors is a major issue in the study of molecular mechanisms governing PCa response to radiotherapy and, in turn, hinders the development of innovative strategies to overcome radioresistance^31,32^.

Genomic rearrangements of chromosome 21 driving ERG expression are among the most common molecular alterations in human PCa and characterize approximately 50% of patients^24^. In particular, 3 Mb deletion in the *q*-arm of human chromosome 21 determines the fusion between *TMPRSS2* exon 1 and *ERG* exon 4^33^. By binding TMPRSS2 promoter, AR induces ERG_Ex4_ transcription in prostate epithelial cells and, in turn, the expression of a N-terminal truncated form of ERG (ERG_Met40_), which preserves transcriptional activity. Even though TMPRSS2-ERG rearrangement is considered an early event in human prostate tumorigenesis, several in vitro and in vivo studies exclude functional roles of ERG in PCa onset but associate ERG activity to tumor progression by providing cancer cells with migratory and invasive molecular competences^34–36^. In a large number of the ERG positive PCa, impairment of PTEN tumor suppressive functions is considered the determining event driving the tumorigenic process^34–37^.

To model such a scenario, both RWPE-1 and RWPE-1 M8 cell lines have been genetically engineered with a viral vector allowing the doxycycline inducible expression of ERG_Met40_ alone or in combination with shRNAs against PTEN (PTEN-KD) (Supplementary Fig. S6a). Upon 48 h of 1 µg/ml doxycycline administration, ERG_Met40_ (hereinafter ERG) expression (Supplementary Fig. S6b, c) drives a significant upregulation of classical ERG target genes in RWPE-1 cells (Supplementary Fig. S6d). Concomitantly, different levels of PTEN downregulation and AKT activation in RWPE-1 cells are achieved through the expression of two independent shRNAs (Supplementary Fig. S6c). From a phenotypic point of view, ERG expression alone is not sufficient to: (i) confer proliferative advantages (Supplementary Fig. S6e); (ii) bypass growth inhibition by cell contact (Supplementary Fig. S6f); or (iii) support cell growth in soft agar (Supplementary Fig. S6g). However, as previously reported^34^, ERG confers migratory and invasive potential to the RWPE-1 cells (Supplementary Fig. S6h–j). On the other hand, RWPE-1 cells experiencing ERG expression in combination with PTEN downregulation show: (i) proliferative advantages (Supplementary Fig. S6e); (ii) bypass of growth inhibition by contact (Supplementary Fig. S6f); (iii) growth in soft agar (Supplementary Fig. S6g); and (iv) marked migratory and invasive propensity (Supplementary Fig. S6h–j), thus recapitulating the typical behavior of malignant cells^35–38^.

Radiation therapy (RT) is the first-line treatment for ~50% of all patients diagnosed with non-metastatic PCa^32^. Of these, 15% is affected by high-risk/locally advanced disease at presentation and has increased risk of tumor recurrence and dying from PCa^3^.

**Fig. 5:**
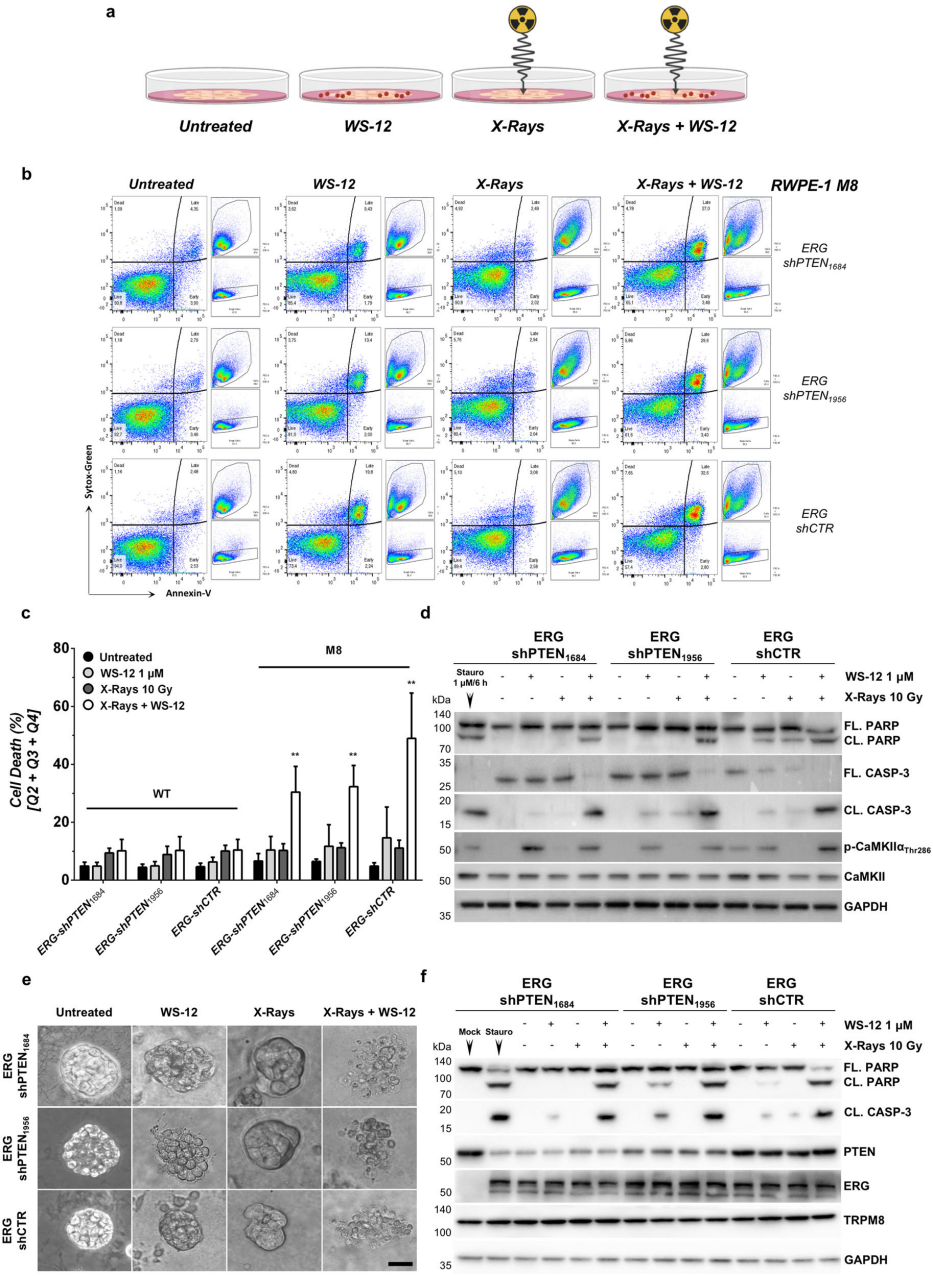
Apoptotic response to X-rays, WS-12 or their combination in RWPE-1 based models of premalignant and malignant prostate lesions. **a** Diagram of the experimental design. Genetically engineered RWPE-1 cells were treated as indicated (BioRender.com). **b** Representative flow cytometry analysis of cell death in ERG + and ERG + /PTEN-deficient RWPE-1 M8 cells treated with WS-12 (1 μM, 12 h), X-rays (10 Gy) or the combination of both. Untreated cells were used as control. **c** Cell death quantification of RWPE-1 M8 cells treated as described in **b** is reported as percentage of total cells. **d** Western blotting analysis of samples described in **b** shows CaMKIIα activation (phosphorylation of Thr286) following WS-12 treatment of RWPE-1 M8 and the classical molecular signature of apoptotic cell death (Caspase-3 and PARP cleavage) in RWPE-1 M8-based premalignant (ERG-shCTR) and malignant (ERG + shPTEN) models of human prostatic disease treated with X-rays plus WS-12. **e** Representative ERG+ and ERG+/PTEN-deficient RWPE-1 M8 3D prostopheres treated with WS-12 (1 μM, 12 h), X-rays (10 Gy) or the combination of both. Untreated spheroids were used as control. Scale bar, 50 μm. **f** Western blotting analysis of treated prostopheres described in **e** showing classical molecular hallmarks of apoptotic cell death (Caspase-3 and PARP cleavage). Error bars, mean ± SD. Experiments were performed in quadruplicate; data were analyzed using a two-way ANOVA test. **P ≤ 0.01.

Administration of a 10 Gy single dose of photons induces a vast amount of double strands breaks in the genome of RWPE-1 cells (Supplementary Fig. S7a). Nevertheless, 48 h after irradiation FACS and western blot analyses of both premalignant (ERG) and malignant (ERG/PTEN-KD) models for the apoptotic markers Annexin-V, Caspase-3, and PARP show a slight increase of cell death in the treated samples compared to the untreated controls (Fig. 5a–d and Supplementary Fig. S7b).

Of note, regardless the amount of TRPM8, ERG/PTEN-KD RWPE-1 models result minimally sensitive to WS-12 administration (Fig. 5a–d and Supplementary Fig. S7b), thus demonstrating a direct role of ERG, besides PTEN/PI3K deregulation, in the establishment of prosurvival programs opposing calcium cytotoxicity in prostate cells.

Remarkably, combination of X-ray and WS-12 treatments evokes a rapid and massive apoptotic response in both ERG and ERG/PTEN-KD RWPE-1 M8 cells expressing greater amount of TRPM8, while leaving unaltered ERG and ERG/PTEN-KD RWPE-1 cells characterized by endogenous levels of channel (Fig. 5a-d).

Cell-to-cell and cell-to-matrix interactions have been proven to support cancer cell survival upon exposure to different types of cellular stressors^39^. To address this point, X-rays, WS-12, and X-rays *plus* WS-12 efficacy has been compared in ERG and ERG/PTEN-KD RWPE-1 M8 3D prostospheres. Even in this setting, combination of X-rays *plus* WS-12 confirms a more effective apoptotic activity than individual X-ray or WS-12 treatments (Fig. 5e, f).

**Fig. 6:**
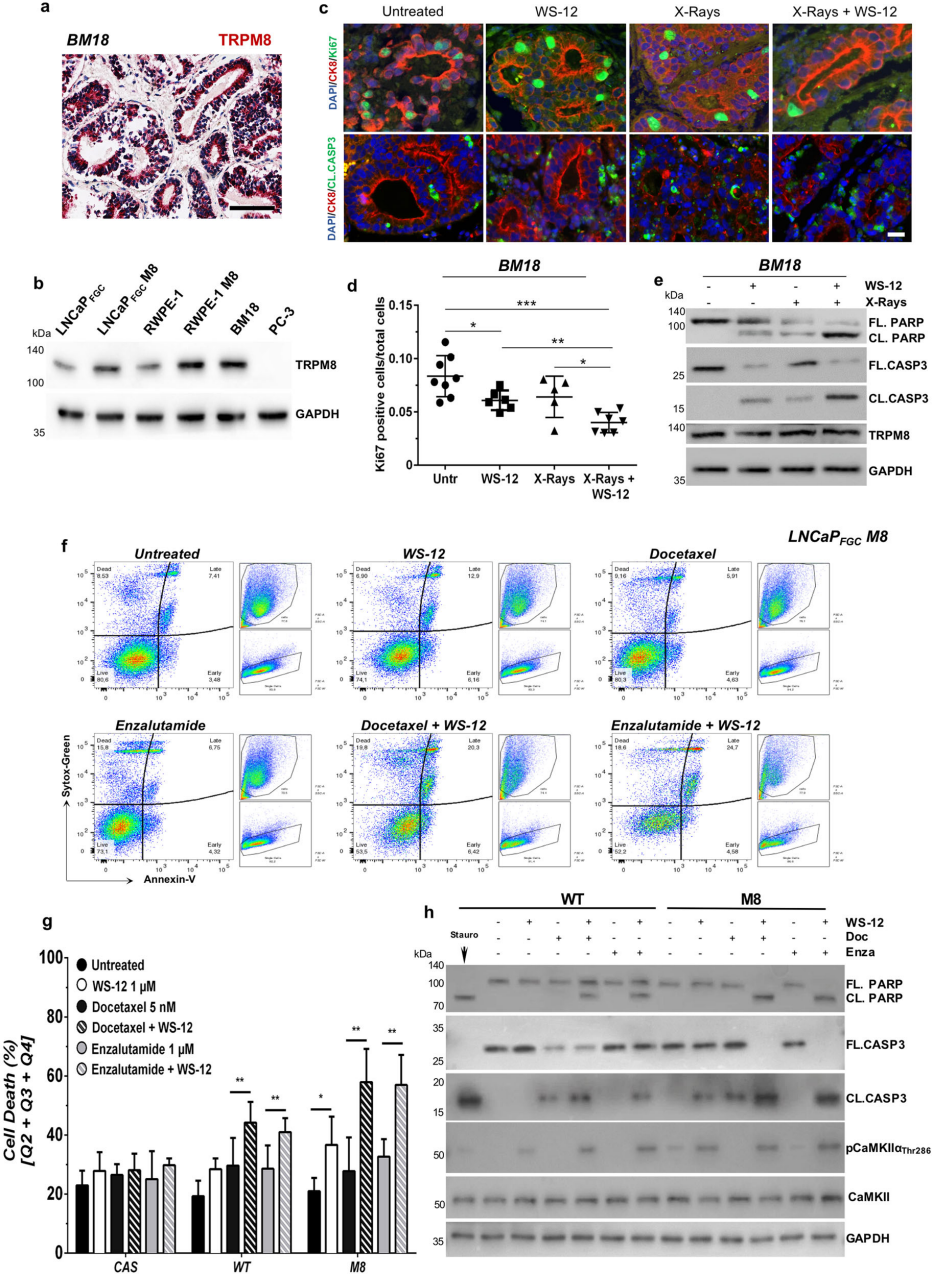
TRPM8 immunoscoring predicts X-rays + WS-12 efficacy. **a** TRPM8 immunostaining of BM-18 PDX. Scale bars, 100 μm. **b** Western blotting analysis shows comparable expression levels of TRPM8 in BM-18 and RWPE-1 M8 cells. **c** Immunofluorescence images showing co-staining of Ki-67 (green, upper panel) or Cleaved Caspase-3 (green, lower panel) with CK8 (red) and DAPI (blue). **d** Percentage of Ki-67 positive cells on a total of 30,000 cells in at least five different areas of the sample. Scale bars, 50 μm. **e** Western blotting analysis in BM-18 PDX tissues slices upon WS-12 (1 μM, 48 h), X-rays (10 Gy), or X-ray + WS-12 treatments showing molecular hallmarks of apoptotic cell death (Caspase-3 and PARP cleavage). Error bars, mean ± SD. Data were analyzed using a two-tailed Student’s *t*-test. *P ≤ 0.05; **P ≤ 0.01; ***P ≤ 0.001. **f** Representative flow cytometry analysis of apoptotic cell death by Annexin-V/Sytox-Green labeling in LNCaP_FGC_ M8 cells treated with WS-12 (1 μM), docetaxel (5 nM), enzalutamide (1 μM), WS-12 + docetaxel, or WS-12 + enzalutamide for 48 h. Untreated cells were used as control. **g** Quantification of dying cells in LNCaP_FGC_ expressing endogenous (WT), increased (M8) or knocked-out (CAS) levels of TRPM8 treated as indicated in **f**. **h** Western blotting analysis of the indicated samples showing CaMKIIα activation (phosphorylation of Thr286) following WS-12 treatment of LNCaP_FGC_ WT and M8 cells and the molecular signature of apoptotic cell death (Caspase-3 and PARP cleavage) upon treatment with combination of WS-12 with docetaxel or enzalutamide. Error bars, mean ± SD. Experiments were performed in quadruplicate; data were analyzed using a two-way ANOVA test. **P ≤ 0.01.

Finally, to further strength our results, we have tested the different treatments in a patient-derived xenograft (PDX BM-18) originally established from a hormone naïve bone metastasis^40^. TRPM8 immunostaining scores 3 (very high) in BM-18 (Fig. 6a) and western blotting analysis demonstrates comparable amounts of full-length TRPM8 protein between BM-18 and RWPE-1 M8 cells (Fig. 6b). By adopting a recently developed ex vivo/in vitro culture system, which supports the survival of normal and tumor human samples for several days^41,42^, we have tested the efficacy of combining X-rays with WS-12 on BM-18 (see Materials and Methods for details). Accordingly, BM-18 slices have been treated with X-rays (10 Gy), WS-12 (1 μM), X-rays + WS-12 or left untreated to serve as control, and, 48 h later, harvested and processed for immunostaining analyses and western blotting studies. In line with our findings, X-rays *plus* WS-12 treatment provokes a significant reduction of proliferation combined with the induction of a potent apoptotic program in BM-18, while single treatments show minimal effects (Fig. 6c–e).

Local lymph nodes are frequently the first site where cancer cells seed once spread outside the prostate. Accordingly, the hormone naïve lymph node metastatic prostate cell line LNCaP_FGC_ represents a valuable proxy where studying the effect of TRPM8 activation on the efficacy of standard *adjuvant* therapies.

Of note, LNCaP cells are *PTEN*-null and express *ETV1*, an additional member of the oncogenic ETS transcription factor family that is frequently overexpressed in TMPRSS2-ERG-negative PCa^43–45^. Intracellular calcium concentration ([Ca^2+^]_i_) has been measured in LNCaP_FGC_ cell lines expressing endogenous (LNCaP_FGC_) or increased (LNCaP_FGC_ M8) amounts of the channel (Fig. 2) upon treatment with different TRPM8 agonists (Supplementary Fig. S8a, b). Exposure of both LNCaP_FGC_ and LNCaP_FGC_ M8 cell lines to menthol (1 mM), WS-12 (1 μM), or icilin (10 μM) fails to prompt any measurable [Ca^2+^]_i_ increases in the totality of cells tested (Supplementary Fig. S8a, b). Further emphasizing a flawed response of both cell lines to TRPM8 activation, western blot analysis of LNCaP_FGC_ and LNCaP_FGC_ M8 cells treated for 12 hours with the potent TRPM8 agonist WS-12 shows no signs of CaMKIIα phosphorylation on Threonine 286 (Supplementary Fig. S8c), while FACS analysis for the apoptotic marker Annexin-V shows comparable rates of cell death in untreated and WS-12 treated LNCaP_FGC_ and LNCaP_FGC_ M8 cells (Supplementary Fig. S8d). However, 48 h treatment with WS-12 induces CAMKIIα phosphorylation on Thr286 in LNCaP_FGC_ and, even more, in LNCaP_FGC_ M8 (Fig. 6h), and almost double the percentage of cell death in LNCaP_FGC_ M8 cells compared to controls (Fig. 6f–h and Supplementary Fig. S9).

Then, LNCaP_FGC_, LNCaP_FGC_ M8, and LNCaP_FGC_ CAS lines have been used to test the efficacy of docetaxel and enzalutamide, two of the most relevant drugs for the treatment of advanced PCa. Forty eight hours treatment with either docetaxel (5 nM) or enzalutamide (1 μM) minimally affect cell viability of LNCaP_FGC_, LNCaP_FGC_ M8, and LNCaP_FGC_ CAS lines. FACS analysis for Annexin-V shows slight differences between treated and untreated samples (Fig. 6f, g and Supplementary Fig. S9a, c), while western blotting highlights Caspase-3 cleavage only upon docetaxel treatment (Fig. 6h and Supplementary Fig. S9b).

Contrariwise, combination of WS-12 with either docetaxel or enzalutamide significantly enhances the rate of cell death in LNCaP_FGC_ cells compared to single drug treatments (Fig. 6f, g and Supplementary Fig. S9c). Beyond the expectations, both combinations rise the percentage of cell death to almost 60% in LNCaP_FGC_ M8 cells expressing greater amount of TRPM8 (Fig. 6f, g). Western blot analyses confirm the activation of apoptotic cell death programs upon either WS-12 *plus* docetaxel or WS-12 *plus* enzalutamide treatments in both LNCaP_FGC_ and LNCaP_FGC_ M8 lines, with LNCaP_FGC_ M8 cells experiencing the most severe responses (Fig. 6h). Caspase-3 and PARP status fully confirms the lack of synergy between WS-12 and chemo/hormone treatments in LNCaP_FGC_ CAS cells (Supplementary Fig. S9b).

## Discussion

Radiation therapy (RT) is a main treatment for patients diagnosed with non-metastatic PCa^32^. The 5-year overall survival milestone is achieved by the vast majority of patients with low aggressive organ-confined tumors; however, patients affected by high-risk/locally advanced PCa at presentation have increased risk of dying from the disease.

Correlation between ionizing radiation (IR) dose and biochemical disease control (but not overall survival) recommends a high-dose approach for the treatment of locally advanced PCa, which, however, leads to toxicity in several organs of the pelvis^46,47^. Parallel to innovative technologies such as intensity modulated radiation therapy (IMRT) and image guided radiation therapy (IGRT) that have substantially reduced the volume of radiation delivered to both gastro-intestinal (GI) and genito-urinary (GU) systems^48,49^, a great effort has been dedicated to the development of radiosensitizers favoring the DNA damage-dependent cancer cell killing activity of IR.

**Fig. 7:**
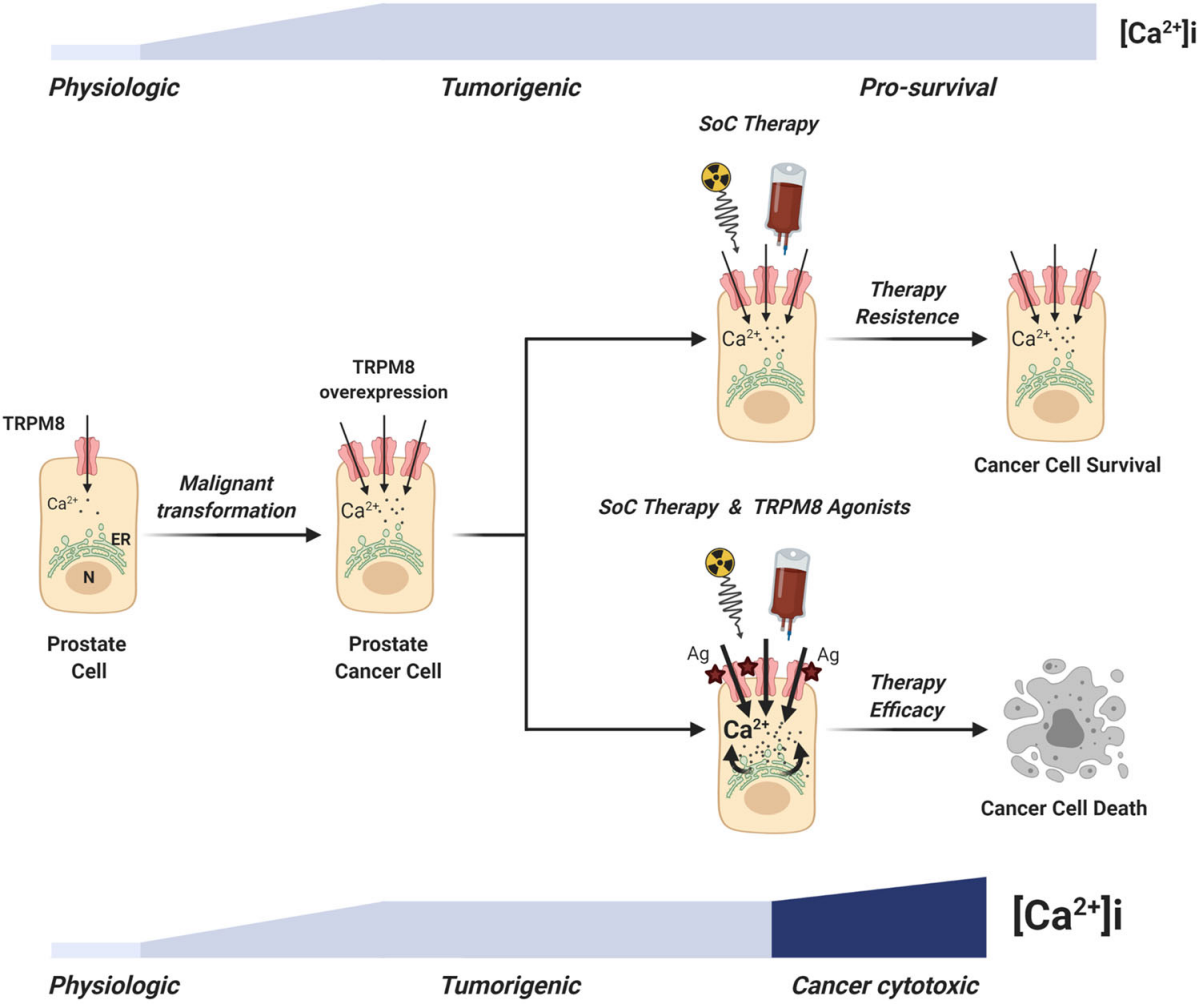
Proposed model for therapy resistance bypass in PCa cells. The scheme shows the lethal synergy between standard-of-care therapies and Ca^2+^ cytotoxicity induced by potent TRPM8 agonists in PCa cells expressing increased amounts of the channel (BioRender.com).

Here, we demonstrate that targeted dysregulation of calcium homeostasis in prostate cancer cells can be an actionable venue to improve the efficacy of standard-of-care therapies for locally advanced/high-risk tumors (Fig. 7).

In prototypes of primary and metastatic PCa recapitulating high TRPM8 expression, Ca^2+^ cytotoxicity induced by potent TRPM8 agonists combined with a sublethal dose of X-rays generates an overwhelming cellular stress that overcomes the anti-apoptotic barriers established in cancer cells by the impairment of PTEN/PI3K/AKT axis. Still, the metastatic progression of the disease is frequently associated with complete loss of PTEN in prostate tumor cells^50^, which has been demonstrated to counteract Ca^2+^-dependent cell death by favoring the proteasome degradation of IP3R3^9^. In the PTEN-null hormone naïve lymph node metastatic prostate cell line LNCaP^43^, Vanden Abeele and colleagues describe the IP3R1 as the IP3 receptor preferentially expressed, followed by almost three times lower levels of IP3R3 and very low, if any, expression of IP3R2^51^. Dysfunctional IP3Rs make TRPM8 agonists unable to induce a rapid and massive Ca^2+^ store depletion in LNCaP, which is the cornerstone of Ca^2+^-induced cytotoxicity. However, different by icilin that induces extensive desensitization of the channel, TRPM8 activation through either menthol or WS-12 is followed by moderate channel adaptation^29,52,53^. This important aspect regarding TRPM8 pharmacology allows us to extend the analysis to longer time points and demonstrate that 48 h of WS-12 treatment doubles the percentage of apoptotic cells in LNCaP_FGC_. This result together with the phosphorylation of CaMKII on threonine 286 suggests that prolonged pharmacological activation of TRPM8 can trigger cytotoxicity, possibly via a smoothly graded increase of [Ca^2+^]_i_ in LNCaP_FGC_ cells, which is likely contributed by continual quantal releases of Ca^2+^ from the ER^54–58^.

Overall, these data demonstrate that extracellular and/or intracellular stimuli leading to minimal increases of [Ca^2+^]_i_ can still determine an harmful cellular stress if prolonged over time. Indeed, 48 h of WS-12 treatment combined with either docetaxel or enzalutamide rises the percentage of cell death in LNCaP_FGC_ cells overexpressing the channel from roughly 20% with single treatments to almost 60%.

In conclusion, our study together with the recently published high-resolution molecular structure of TRPM8^59,60^, the development of TRPM8 agonists with improved pharmacological characteristics^61^, and innovative methods to deliver TRPM8 agonists to PCa cells^62^, support the design of preclinical in vivo trials for testing safety^63^ and efficacy of TRPM8 activation as a novel strategy for a more effective treatment of locally advanced/high-risk PCa patients, an oncologic population with urgent unmet needs.

## Materials and methods

### Analysis of TRPM8 expression levels using RNA-seq data

Landscape of TRPM8 transcript levels in normal and primary tumor samples across different tissues was retrieved from^24,64^. RPKM levels were computed and intersample quantile normalization was performed. RNA-seq analysis of TRPM8 and other genes in prostate cancer samples was conducted using previously published data^24–26^. AR signaling scores were retrieved from previous analysis^25^. AR signaling scores measure the Pearson’s correlation coefficient of the transcript level of 30 genes against a reference sample^25^. Normalized RNA-seq counts for TRPM8 isoforms were obtained from TCGA Legacy Archive (https://portal.gdc.cancer.gov/legacy-archive). Association of high TRPM8 transcript level with OS (Overall Survival), DFI (Disease-Free Interval) and PFI (Progression-Free Interval) in the TCGA PCa dataset was performed using data reported in ref. ^65^. TRPM8 transcript level was considered high when greater or equal than the 75th percentile of overall TRPM8 distribution across all TCGA patients (*N* = 497). Analysis was performed using Keplan-Meier estimator and Likelihood Ratio (LR) test statistics.

### Cell lines

The human cell lines were purchased from the ATCC and cultured according to the manufacturer’s instructions in a humidified incubator at 37°C and 5% CO_2_. Cell lines were tested for specific markers by WB and RT-qPCR and routinely checked for *Mycoplasma* (MycoAlert Kit, Lonza). For 3D cell cultures, RWPE-1 cells were seeded in KSFM medium supplemented with 5% of Matrigel^TM^ (Corning) and incubated at 37 °C for 8 days. Prostospheres growth was imaged using a Leica DFC 450 C microscope.

### Real-time quantitative PCR

Total RNA was extracted using Direct-zol™ RNA MiniPrep kit (Euroclone) and the purified RNA was reverse-transcribed using iScript™ cDNA synthesis Kit (Biorad) according to the manufacturer’s instructions. Each qRT-PCR was performed in triplicate on a CFX96 qPCR Thermal cycler (Biorad) and the results were normalized to GAPDH and TBP mRNA levels. The specific primers sequences used are provided in Supplementary Table S3.

### Western blot

Cells were lysed with RIPA buffer supplemented with protease and phosphatase inhibitors. Protein samples were subjected to SDS-PAGE and transferred to PVDF membranes (Hybond^TM^, Fisher Scientific). The antibodies used are provided in Supplementary Table S4. Immunoblots were revealed using the ECL Select WB Detection Reagent (GE Healthcare) and an Alliance LD2 system (UVITEC). WB were performed in at least three independent biological replicates; representative data are shown.

### Irradiation

Six wells plates with 70% confluent cells were irradiated with a X-ray beam (single dose of 10 Gy) by using the Xstrahl RS225 X-ray research irradiator cabinets at the Trento Institute for Fundamental Physics and Application (TIFPA) at a dose rate of 1 Gy/min at RT. Following irradiation, cells were treated as indicated in the figure and postincubated at 37 °C for 4 h (immunofluorescence microscopy) or 12–48 h (FACS and WB analysis).

### Immunofluorescence

Cells were grown on coverslips, fixed with 4% PFA and permeabilized in 5% FBS/0.1% Triton X-100. Afterward, cells were blocked and incubated with primary antibodies overnight at 4°C (see Supplementary Table S4). After washing, cells were incubated with Alexa Fluor conjugated secondary antibodies and counterstained with DAPI. For the semiquantitative analysis of TRPM8 expression, we adopted a nonpermeabilizing protocol to detect only its localization at the plasma membrane. Cells grown on coverslips were washed with ice-cold 2% BSA in PBS and incubated with anti-TRPM8 (Alomone Labs, ACC-049) and anti-E-Cadherin antibodies for 1 h at 4 °C. After washing, cells were incubated with Alexa Fluor conjugated secondary antibodies, fixed with 4% PFA and counterstained with DAPI. For the quantification of plasma membrane associated TRPM8 channels, TRPM8 dots from more than 6000 cells were counted by two independent researchers. All the images were acquired using a Leica DM6000 CS confocal microscope. Immunofluorescence studies were performed in at least three independent biological replicates; representative data are shown.

### FACS analysis

Cells were cultured at about 60% confluence in six-well dishes and treated and/or irradiated as indicated in the figure. After 12–48 h, cells were pelleted with the exhausted medium and washed in 1X Binding Buffer solution. Cell death and apoptosis rates were determined with Annexin-V-FITC (BD Biosciences) or Annexin-V-APC (Life Technologies) and 7-AAD (ThermoFisher Sci) or SYTOX^TM^-Green Nucleic Acid stain (Life Technologies), staining according to manufacturer’s instructions. For FACS analysis a CantoA flow cytometer (BD Biosciences) was used and data were analyzed with FlowJo software (Treestar). FACS analyses were performed in at least three independent biological replicates; representative data are shown.

### Fluorescence calcium imaging

Calcium-imaging experiments were performed as previously described^66^. Cells were perfused with the medium with a microperfusion system in the absence or presence of menthol, WS-12 or icilin. Fluorescence intensity values were converted in Ca^2+^ concentrations assuming a Kd of 224 nM^67^.

### Electrophysiology

Macroscopic currents from RWPE-1 stably overexpressing TRPM8 cells were recorded at room with an Axopatch 200B amplifier (Molecular Devices, Union City, CA), using the whole-cell configuration of the patch-clamp technique, as previously described^61,68^.

### Ex vivo/in vitro culture system

Ex vivo model with tissue slices obtained from intact BM-18 patient-derived xenograft (PDX) maintained in adult male CB17/SCID mice^40^, was performed as described previously^41,42^. Briefly, for single compound (WS-12, 1 μM), X-rays treatment (10 Gy), combination and untreated control, tissue slices were placed on a nitrocellulose membrane in contact with culture medium, oxygenated and maintained at 37 °C for 48 h. Tissues were recovered and subjected to WB analysis or fixed with 4% PFA and embedded in paraffin. In vivo experiment for PDX collection and maintenance was performed according to the directions of the ethical committee for animal studies of Canton Bern, Switzerland (Protocol number BE12/17).

### Immunohistochemistry

Cell lines were grown at confluence in 10 cm Petri dishes, trypsinized and collected by centrifugation. Pellets were fixed overnight in 4% PFA at 4 °C, dehydrated with alcohols and embedded in paraffin. Paraffin blocks of cell pellets and BM-18 were cut at the microtome to obtain 5-µm thick sections, which were recovered on glass slides, deparaffinized, and used for immunohistochemistry. Immunohistochemical analysis was performed at the Department of Histopathology (S. Chiara Hospital, Trento, Italy) using an automatic immunostainer (BOND-III platform, Leica Biosystems). Antigen retrieval was carried out with optimized BOND reagents (Bond epitope retrieval solution 1, Leica Biosystems) at pH 6. The following primary antibodies were used: TRPM8 (Alomone Labs, ACC-049) at 1:300 dilution and Cytokeratin HMW (34βE12) (PA0134, Leica Biosystems) optimally diluted for use (see also Supplementary Table S4). BOND compact polymer detection solution (Leica Biosystems) was used for the detection. High-resolution images were acquired using an Axio Imager M2 microscope (Zeiss). A prostate adenocarcinoma tissue microarray (TMA), containing 58 cases of adenocarcinomas and 6 normal tissues (192 total cores, triplicate cores per case), was purchased from US Biomax, Inc. (PR208a, see Supplementary Table S5). Samples histology and intensity of TRPM8 immunostaining were reviewed by a trained pathologist (M.B.) to ensure appropriate assignment of the following scores: weak (0), moderate (1), high (2), and very high (3). Deidentified primary and lymph node metastatic human PCa matched samples (*n* = 6) were retrieved from the tissue bank archives of the Surgical Pathology Unit of the S. Chiara Hospital (Trento, Italy) upon approval of the Hospital ethical committee (Prot.:1946 I.D.:112786962). TRPM8 stained histologic slides were reviewed independently by two trained pathologists (M.B. and F.G.C).

### Statistical analysis

Statistical comparison of TRPM8 transcripts levels across sample’s classes was performed using a two-tailed Wilcoxon–Mann–Whitney test with a significance level set at 5%. Correlation between *TRPM8* transcript levels, transcript levels of *NK3X-1* and *KLK2* and *AR* score was performed using Pearson correlation statistics with a significance level set at 5%. GraphPad Prism 6 software (GraphPad Software Inc.) was used for all statistical analyses applied to the experimental data. Student’s *t*-test or one-way ANOVA were used for comparison between two groups, while two-way ANOVA was used to compare the magnitude of changes among different conditions in more than two groups. Data are presented as mean ± SD of at least three independent experiments. *P* value < 0.05 was considered as statistically significant (*P < 0.05; **P < 0.01; ***P < 0.001).

## Supplementary information

Supplementary Front Page

Supplementary Material & Methods

Supplementary Figure Legends

Supplementary Figure S1

Supplementary Figure S2

Supplementary Figure S3

Supplementary Figure S4

Supplementary Figure S5

Supplementary Figure S6

Supplementary Figure S7

Supplementary Figure S8

Supplementary Figure S9

Supplementary Tables S1-S5

## Data Availability

All the data generated or analyzed during this study are included in this article and its supplementary information files or available from the author upon reasonable request.
