# Supplementary material for: Calcium cytotoxicity sensitizes prostate cancer cells to standard-of-care treatments for locally advanced tumors
Source: Cell Death Dis. 2020 Dec 7;11(12):1039. doi: 10.1038/s41419-020-03256-5 (PMC7721710; doi:10.1038/s41419-020-03256-5)
Supplement: Supplementary file 1 — Supplementary Front Page [file 41419_2020_3256_MOESM1_ESM.pdf]

Calcium cytotoxicity sensitizes prostate cancer cells to standard-of-care treatments for locally advanced tumors

Alessandro Alaimo[1], Marco Lorenzoni[1], Paolo Ambrosino[2], Arianna Bertossi[1], Alessandra Bisio[1], Alice Macchia[1], Eugenio Zoni[3], Sacha Genovesi[1], Francesco Cambuli[1], Veronica Foletto[1], Dario De Felice[1], Maria Virginia Soldovieri[4], Ilaria Mosca[4], Francesco Gandolfi[1], Matteo Brunelli[5], Gianluca Petris[1], Anna Cereseto[1], Alvaro Villarroel[6], George Thalmann[3,7], Francesco Giuseppe Carbone[8], Marianna Kruithof-de Julio[3,7], Mattia Barbareschi[8], Alessandro Romanel[1], Maurizio Taglialatela[9], and Andrea Lunardi[1]

[1]Department of Cellular, Computational and Integrative Biology (CIBIO), University of Trento, Trento, Italy.

[2]Department of Science and Technology (DST), University of Sannio, Benevento, Italy.

[3]Department for BioMedical Research, Urology Research Laboratory, University of Bern, Bern, Switzerland.

[4]Department of Medicine and Health Sciences, University of Molise, Campobasso, Italy.

[5]Department of Pathology AOUI, University of Verona, Verona, Italy.

[6]Biofisika Institute (CSIC, UPV/EHU), University of the Basque Country, Leioa, Spain.

[7]Department of Urology, Inselspital, Bern University Hospital, University of Bern, Bern, Switzerland.

[8]Unit of Surgical Pathology, Santa Chiara Hospital, Trento, Italy.

[9]Department of Neuroscience, University of Naples "Federico II", Naples, Italy.

Corresponding author

Andrea Lunardi, Armenise-Harvard Laboratory of Cancer Biology & Genetics, Department of Cellular, Computational and Integrative Biology (CIBIO), University of Trento, Via Sommarive 9 Povo Trento, 38123, Italy. Phone: 39-0461-285288; E-mail: andrea.lunardi@unitn.it

Supplementary materials

Supplementary Methods

Supplementary Figure Legends

Supplementary Fig. S1: TRPM8 expression in human PCa datasets.

Supplementary Fig. S2: TRPM8 immunostaining in PCa**.**

Supplementary Fig. S3: TRPM8 expression correlates with AR activity.

Supplementary Fig. S4: Characterization of AR expression and activity in RWPE-1 cell line.

Supplementary Fig. S5: RWPE1 M8 response to TRPM8 agonists icilin, menthol and WS- 12.

Supplementary Fig. S6: Modeling aggressive primary PCa in RWPE-1.

Supplementary Fig. S7: Radiotherapy resistance PCa models.

Supplementary Fig. S8: TRPM8 activity in hormone naïve metastatic $LNCaP_{FGC}$ cells.

Supplementary Fig. S9: TRPM8-null $LNCaP_{FGC}$ response to treatment.

Supplementary Table S1: Sequence of shRNAs

Supplementary Table S2: sgRNAs and TRPM8 target sequences

Supplementary Table S3: Description of primers used in this study

Supplementary Table S4: List of antibodies used in this study

Supplementary Table S5: Prostate cancer tissue microarray
