# Supplementary material for: Calcium cytotoxicity sensitizes prostate cancer cells to standard-of-care treatments for locally advanced tumors
Source: Cell Death Dis. 2020 Dec 7;11(12):1039. doi: 10.1038/s41419-020-03256-5 (PMC7721710; doi:10.1038/s41419-020-03256-5)
Supplement: Supplementary file 2 — Supplementary Material & Methods [file 41419_2020_3256_MOESM2_ESM.pdf]

Supplementary Material and Methods

Generation of plasmid vectors and stable cell lines

The coding sequence of human TRPM8 (NM_024080) was PCR amplified from $LNCaP_{FGC}$ cells. PCR products were cloned into the lentiviral vector pAIB. To produce viral particles, 50% confluent HEK-293T cells were co-transfected with 10 µg of pAIB-TRPM8, 2.5 µg of the envelope pHDM-VSVG, 7.5 µg of the packaging psPax2 plasmids and 50 µl of polyethylenimine (PEI). Lentivirus was harvested 48 h post-transfection and 5 µg/ml polybrene was added. Subconfluent RWPE-1 and $LNCaP_{FGC}$ cells were infected with lentivirus and were selected adding 10 µg/ml blasticidin.
To obtain RWPE-1 cells inducible for the expression of ERG and shRNA molecules against PTEN, we started from two pTGMP plasmids (Addgene #32716) harboring miR30-based shRNA sequences targeting PTEN and one containing the sequence targeting Luciferase which were generated as follow. The miR30-based shRNA sequences targeting PTEN (sh1684h and sh1956h) were taken from doi: 10.1038/nature13239, whilst the sequence targeting Luciferase (shCRT) was obtained by the pENTR/pTER shLUC plasmid (Addgene #17472). The 97-mer miR30-shRNA inserts were amplified by PCR (see primers in Supplementary Table S1), and cloned into the pTGMP. ERG coding sequence was amplified by PCR from VCaP cells. The N-terminal truncated form of ERG, ERG_{Met40} (Exons 4-11, variant 2, NM_004449.4), was confirmed by sequencing. The PCR product was cloned into pTGMP-shPTENs and pTGMP-shCRT obtaining pTGMP-ERG-shRNA vectors. Finally, the cassette Ubc-rtTA3-IRES was PCR amplified from pINDUCER10 (Addgene #44011) and inserted into the pTGMP-ERG-shRNA vectors. To produce retroviral particles and for retroviral infection of RWPE-1 cells, the same protocol described above was used except for the packaging that was pRetro-Gag-Pol plasmid. Stable cells were selected using puromycin (1 µg/mL, for RWPE-1 WT cells) or puromycin (1 µg/mL) and blasticidin (10 µg/mL, for RWPE-1 M8 cells). The inducible expression of ERG and shRNA against PTEN (or shCRT) was stimulated adding doxycycline (1 µg/ml) to the medium for at least 48 h.
To obtain RWPE-1 AR stable cell line, full-length human AR cDNA was cloned into pAIP vector (Addgene #74171), packaged into lentiviral particles in HEK293T cells and used to infect RWPE-1 cells. Stable cells were selected using puromycin (1 µg/ml).

Generation of the RWPE-1 and $LNCaP_{FGC}$ TRPM8KO cell lines

To generate TRPM8 Knock Out (CAS) cells, four sgRNAs were designed using the Broad Institute GPP Web Portal tool (https://portals.broadinstitute.org/gpp/public/analysis-tools/sgrna-design). The sgRNAs and corresponding TRPM8 target sequences are provided in Supplementary Table S2. pL-CRISPR.EFS.GFP plasmid (Addgene #57818) was used to express Cas9, sgRNAs and GFP. Primers were annealed and cloned into pL-CRISPR.EFS.GFP plasmid similarly to previously described. RWPE-1 and $LNCaP_{FGC}$ cells were transfected with 2 µg of each sgRNA by electroporation. After 48 h, cells were enriched by FACS sorting on a FACS Aria II cell sorter (BD Biosciences) to isolate those expressing GFP. The sorting buffer was 2% FBS, 2X Pen/Strep, 1 mM EDTA in PBS. Cells were grown and tested for TRPM8 silencing by WB.

Chemicals and drugs

L-Menthol, WS-12, Docetaxel, Staurosporine, Ionomycin and DHT were purchased from Sigma, Icilin from Alomone Labs and Enzalutamide from Cayman chemicals. In each experiment, the same volume of solvent used for tested molecules was added to the control solution.

Small interfering RNA targeted against TRPM8

Cells were plated on a six-well plate (2×10^5 cells/well) and transfected at 80% confluence with two different siRNAs against TRPM8 (100 nM) or negative control siRNA using Lipofectamine® (Life Tech) as described in the manufacturer's protocol. Cells were harvested for WB analysis at 24 or 48 h. These sense siRNA sequences were tested:
siRNA1 GGUGCUUUGGAUUCUCACGG from Ambion 104796 (Life Tech)
siRNA2 GGAUGCCCUGACAUCUUUCU from Ambion 104798 (Life Tech)
$siRNA_{CRT}$ Silencer® Negative Control #1 from Ambion AM4611 (Life Tech)

Subcellular fractionation

Cell fractionation was performed using NE-PER Nuclear and Cytoplasmic Extraction Kit (ThermoFischer Sci) according to the manufacturer's instructions. Samples were subjected to Western blot analysis.

Growth proliferation assay

Cells were plated in 12-well plate (1×10^4 cells/well). Plates were collected on day 0, 2, 4, 6 and 7. Cells were washed with PBS, fixed with 10% formalin (Sigma), washed again with PBS and stained with 0.1% Crystal Violet (Sigma) solution (in 20% methanol) for 30 min. Afterward, cells were washed with dH_20, dried, and Crystal Violet was extracted with 10% acetic acid for 30 min. Finally, absorbance was measured at 595 nm.

Colony formation

About 500 cells were seeded in six-well plates and cultured at 37°C. After two weeks, the colonies were washed, fixed with 10% formalin, and stained with 0.1% Crystal Violet solution. Colonies were washed, dried and imaged with a Chemidoc XRSF (Biorad).

Soft agar colony formation assay

The anchorage-independent growth assay was achieved using soft agar as a supporting matrix. First, a 0.7% base layer of agarose in warm KSFM medium was placed in each well of six-well plates and allowed to solidify. The cells (2×10^4 per well) in 0.35% agarose containing KSFM media were plated as the top layer. Plates were incubated at 37°C and medium was changed twice a week. After 3 weeks, colonies were stained with a solution of 0.01% Crystal Violet, washed and then imaged with a Leica MZ16F stereomicroscope.

Wound healing assay

Cells were cultured to confluence in six-well plates at 37°C. A 200 µl pipette tip was used to scratch the cells monolayer across the wells and images were captured using a Leica DFC 450C microscope after the scratch (0 h) and after 24 and 48 h. The migration rates of the RWPE-1 cells were estimated using ImageJ software (ImageJ 1.46r NIH).

Migration and invasion assays

For transwell cell migration and invasion assays, we used CHEMICON® QCM™ fluorimetric Cell Migration Assay Kit (Merck-Millipore) and CHEMICON® QCM™ fluorimetric Cell Invasion Assay chambers (Merck-Millipore), respectively, following the manufacturer's instructions. Migrated and invaded cells were quantified and reported graphically as bar charts.
