# Supplementary material for: Calcium cytotoxicity sensitizes prostate cancer cells to standard-of-care treatments for locally advanced tumors
Source: Cell Death Dis. 2020 Dec 7;11(12):1039. doi: 10.1038/s41419-020-03256-5 (PMC7721710; doi:10.1038/s41419-020-03256-5)
Supplement: Supplementary file 3 — Supplementary Figure Legends [file 41419_2020_3256_MOESM3_ESM.pdf]

Supplementary Figure Legends

Supplementary Fig. S1 TRPM8 expression in human PCa datasets. a-b TRPM8 mRNA expression in benign prostate tissue (N), primary tumor (PCa) and castration resistant metastatic (CRPC-Met) samples retrieved from Grasso (**a**) and Taylor (**b**) microarray expression datasets. **c** Analysis of TRPM8 mRNA isoforms in human prostate specimens defines PM-associated 6TM (full-length, uc002vvh) and ER-associated 4TM TRPM8 (short, uc010fyk) as the only two mRNA isoforms to be expressed. **d** Schematic representation of the 26 exons belonging to the human *TRPM8* gene (upper panel). Exon composition of PM-associated 6TM (full length) and ER-associated 4TM TRPM8 isoform are shown respectively in the middle and lower panels. **e** Expression levels of TRPM8 mRNA in primary PCa stratified according to the Gleason score as retrieved from TCGA RNAseq dataset. **f** Association between patients with high TRPM8 transcript level against OS (Overall Survival, left panel) and DFI (Disease-Free Interval, right panel). Keplan-Meier plots are shown along with the results of Likelihood Ratio (LR) test statistics. Patients with high TRPM8 transcript level (>=75th percentile of overall TRPM8 distribution across all TCGA patients) are shown in black.

Supplementary Fig. S2 TRPM8 immunostaining in PCa. a Immunohistochemical analysis of TRPM8 on sections of in paraffin embedded RWPE-1, RWPE-1 M8, $LNCaP_{FGC}$ and PC-3 cellular pellets. **b** Representative immunostaining for Cytokeratin HMW (HMWCKs) and TRPM8 in human PCa specimens showing greater amount of TRPM8 protein in HMWCKs negative malignant lumens. **c** Representative images of normal prostate tissue and stages I-to-IV PCa cores stained for TRPM8. **d** TRPM8 immunostaining of matched primary PCa (A, B, C, D) and hormone naïve lymph node metastases (a, b, c, d). Scale bar, 10 µm**. e** Left panel, structure of the prostate tissue microarray (58 cases of prostate adenocarcinoma and 6 normal prostate tissues, triplicate cores per case, 192 total cores). Right panel, TRPM8 staining. Scale bars, 100 µm.

Supplementary Fig. S3 TRPM8 expression correlates with AR activity. a End-point PCR analysis showing PM-associated 6TM (full length) and ER-associated 4TM TRPM8 isoforms in both RWPE-1 and $LNCaP_{FGC}$ cell lines. Minimal level of 6TM TRPM8 expression is also detectable in PWR-1E cells. **b** Western blotting analysis with two independent commercially available antibodies detecting full length 6TM TRPM8 protein in both RWPE-1 and $LNCaP_{FGC}$ cell lines, while the ER-associated 4TM TRPM8 shorter variant is visible only in $LNCaP_{FGC}$ cells. **c** RWPE-1, $LNCaP_{FGC}$ and PWR-1E cells were transfected with non-targeting (CTR) or TRPM8 siRNA (siRNA1) as indicated. Antibodies specificity was confirmed by TRPM8 knockdown by immunoblot analysis. **d-e** End-point PCR (**d**) and Western blotting (**e**) analyses showing expression of full-length PM-associated 6TM TRPM8 isoform primarily in hormone sensitive AR-positive metastatic PCa cell lines. With the exclusion of the $LNCaP_{FGC}$ cell line, end-point PCR shows minimal expression of 4TM TRPM8 mRNA isoform in metastatic PCa cell lines. **f** Correlation between TRPM8 expression and AR transcriptional activity in benign prostate tissue, primary PCa and castration resistant metastatic adeno-PCa (upper panel). Middle and lower panels highlight the correlation between the expression levels of TRPM8 and the AR transcriptional targets NKX3.1 and KLK2 (PSA). Results were analyzed using Pearson correlation statistics with a significance level set at 5%.

Supplementary Fig. S4 Characterization of AR expression and activity in RWPE-1 cell line. a AR expression in AR-positive $LNCaP_{FGC}$, RWPE-1 and PWR-1E. AR-negative PC3 and DU-145 prostate cell lines are used as negative control to test the specificity of the two antibodies against AR. **b** Biochemical fractionation of cytoplasm and nucleus (C-N) cell compartments from RWPE-1 and $LNCaP_{FGC}$ cells showing AR nuclear shuttling upon dihydrotestosterone (DHT, 10 nM) treatment. Fibrillarin and β-Tubulin are used as nuclear and cytoplasmic markers, respectively. **c** RT-qPCR showing increased expression of AR target genes *HPGD*, *NFKB1*, *TMPRSS2* in RWPE-1 cells treated with DHT (10 nM) for 48 hours. **d** Western blotting analysis showing expression of AR in RWPE-1 and RWPE-1 AR stably overexpressing the androgen receptor. **e** Immunofluorescence analysis shows the AR nuclear translocation upon DHT administration to RWPE-1 AR cells. Scale

bar, 50 μm. **f** RT-qPCR analysis showing AR target genes *HPGD*, *NFKB1*, *TMPRSS2* increased expression in RWPE-1 AR cells treated with DHT (10 nM) for 48 hours. Experiments were performed in quadruplicate unless differently indicated; data were analyzed using a two-tailed Student's t-test. *, P ≤ 0.05; **, P ≤ 0.01; ***, P ≤ 0.001.

Supplementary Fig. S5 RWPE-1 M8 response to TRPM8 agonists icilin, menthol and WS-12. a FACS analysis for Annexin V/Sytox-Green showing no signs of cell death in two independent TRPM8-null RWPE1 (CAS) lines upon 12 hours of WS-12 (1 μM) treatment. Untreated cells were used as control (CTR). **b** Quantification by FACS of cell death in RWPE1 M8 cells following 12 hours treatment with icilin (10 μM), menthol (1 mM), or WS-12 (1 μM). Untreated cells were used as control. Experiments were performed in triplicate; data were analyzed using a one-way ANOVA test. **, P ≤ 0.01; ***, P ≤ 0.001.

Supplementary Fig. S6 Modeling aggressive primary PCa in RWPE-1. a Schematic representation of doxycycline inducible pTGMP-ERG-shRNA vector. The retroviral system drives the constitutive expression of rtTA3 and the doxycycline inducible expression of a single transcript (ERG) ending with the miR30 cassette containing shRNA sequences. **b** RT-qPCR analysis of ERG expression in genetically engineered RWPE-1 cells upon doxycycline administration (n = 5 independent experiments, statistical analyses were done using a two-tailed Student's t-test, **P ≤ 0.01). **c** Representative Western blot showing ERG expression alone or in combination with different levels of shRNA-mediated PTEN down regulation and AKT activation ($pAKT_{S473}$) in genetically engineered RWPE-1 cells upon doxycycline administration (1 μM, 48 hours). **d** RT-qPCR analysis showing increased expression of ERG targeted genes *PLAT*, *KCNS3*, *ARHGDIB* and *ADAMTS1* in genetically engineered RWPE-1 cells treated with doxycycline (1 μM, 48 hours). Experiments were performed in quadruplicate or quintuplicate; data were analyzed using a two-tailed Student's t-test. *, P ≤ 0.05; **, P ≤ 0.01; ***, P ≤ 0.001. **e-g** Growth curves (**e**), colony formation (**f**) and soft agar (**g**)

assays showing signs of growth advantages conferred by ERG expression plus PTEN deficiency to RWPE-1 cells. **h-j** Wound healing (**h**), transwell migration (**i**) and invasion (**j**) studies demonstrating migratory and invasive behaviors conferred to RWPE-1 cells by ERG expression and further enhanced by PTEN deficiency. Experiments were performed in triplicate; data were analyzed using a one-way ANOVA test. *, $P \leq 0.05$; **, $P \leq 0.01$; ***, $P \leq 0.001$.

Supplementary Fig. S7 Radiotherapy response in PCa models. a γH2AX staining showing diffused DNA double strand breaks in ERG-shPTEN$_{1684}$ RWPE-1 cells following 10 Gy (single dose) X-rays treatment. Scale bar, 50 μm. **b** FACS analysis of cell death response in ERG-shCTR, ERG-shPTEN$_{1684}$ and ERG-shPTEN$_{1956}$ RWPE-1 cell lines expressing endogenous levels of TRPM8 following WS-12 administration (1 μM, 12 hours), X-rays radiation (10 Gy), or a combination of both.

Supplementary Fig. S8 TRPM8 activity in hormone naïve metastatic LNCaP$_{FGC}$ cells. a-b Representative images (upper panels) and traces (lower panels) showing $[Ca^{2+}]_i$ changes in LNCaP$_{FGC}$ cells expressing endogenous (WT) (**a**) or increased (M8) levels (**b**) of TRPM8 upon treatment with control solution (CTR) or menthol (1 mM). Time of menthol exposure is indicated by the bar on top of the traces. Right panels report the quantification of $[Ca^{2+}]_i$ peaks measured upon perfusion of LNCaP$_{FGC}$ WT (**a**) or LNCaP$_{FGC}$ M8 (**b**) cells with different TRPM8 activators. The inset graph indicates the percentage of cells that have responded to the TRPM8 agonists. **c** Western blot analysis of phospho-Thr286 CaMKIIα in LNCaP$_{FGC}$ cells expressing endogenous (WT) or increased levels of TRPM8 (M8) upon WS-12 (1 μM, 12 hours) administration. Ionomycin is used as positive control. **d** FACS analysis of cell death response in LNCaP$_{FGC}$ and LNCaP$_{FGC}$ M8 cells following 12 hours treatment with WS-12 (1 μM). Untreated cells were used as control.

Supplementary Fig. S9 TRPM8-null LNCaP$_{FGC}$ response to treatment. a-b FACS (**a**) and Western blot (**b**) analysis of cell death response in TRPM8-null LNCaP$_{FGC}$ (CAS) cells upon 48 hours of treatment with docetaxel (5 nM), enzalutamide (1 μM), WS-12 (1 μM), docetaxel plus WS-12 or enzalutamide plus WS-12 combinations. Untreated cells were used as control. **c** FACS analysis with Annexin V/syntox-Green of LNCaP$_{FGC}$ expressing endogenous levels of TRPM8 treated as in (**a**).
