# Supplementary figures and images for: Calcium cytotoxicity sensitizes prostate cancer cells to standard-of-care treatments for locally advanced tumors
Source: Cell Death Dis. 2020 Dec 7;11(12):1039. doi: 10.1038/s41419-020-03256-5 (PMC7721710; doi:10.1038/s41419-020-03256-5)

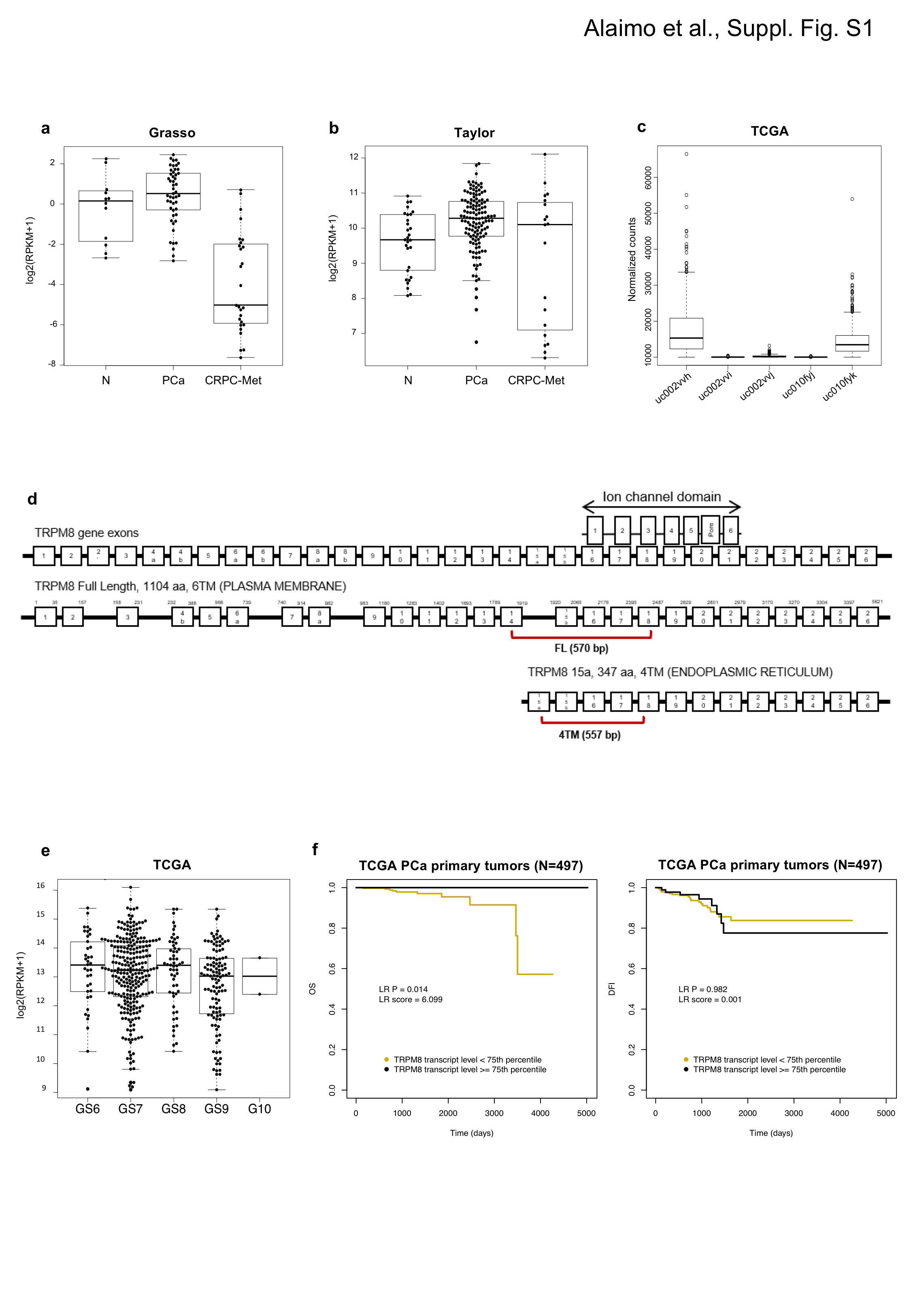

Supplement: Supplementary file 4 — Supplementary Figure S1 [file 41419_2020_3256_MOESM4_ESM.tif]

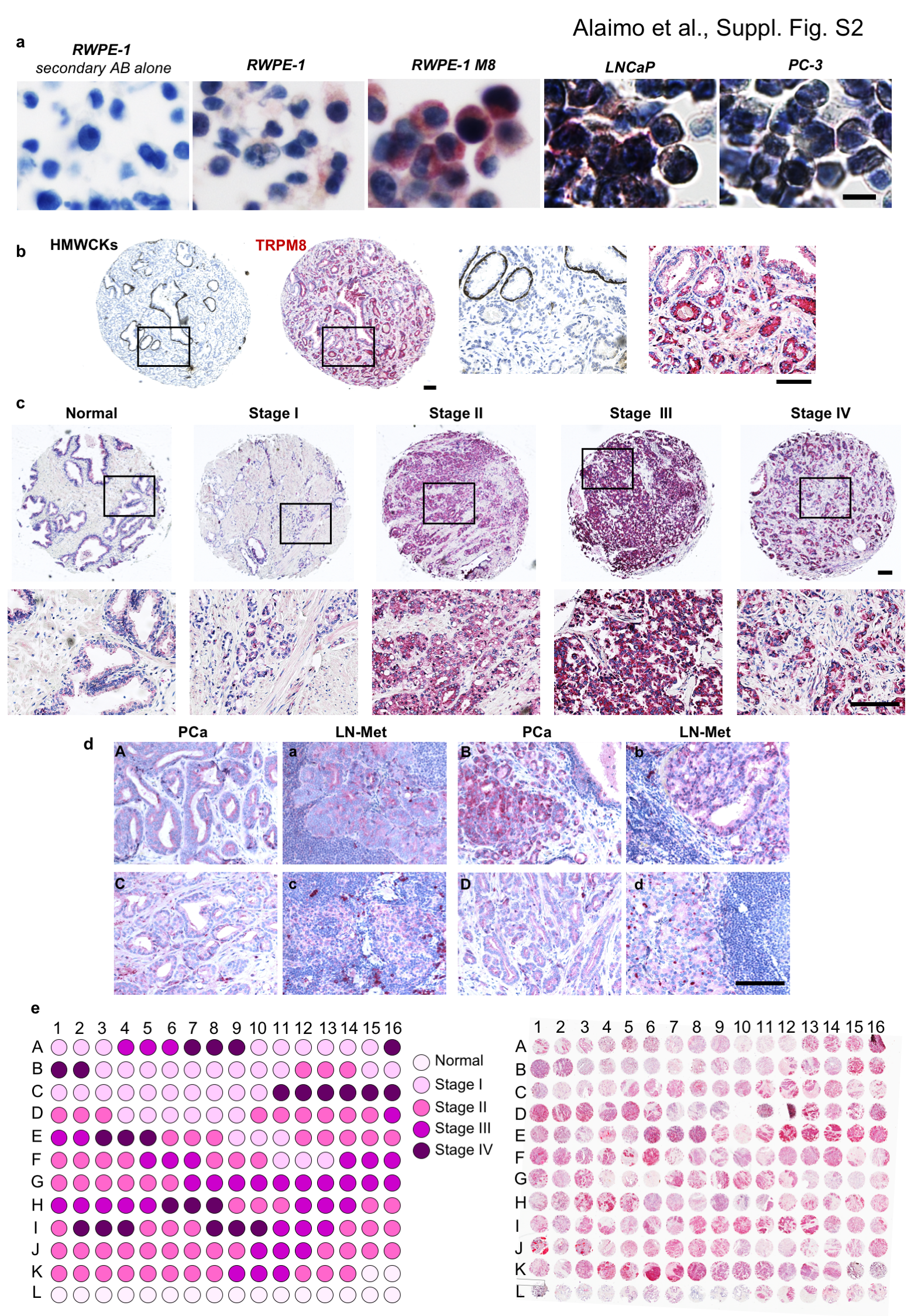

Supplement: Supplementary file 5 — Supplementary Figure S2 [file 41419_2020_3256_MOESM5_ESM.tif]

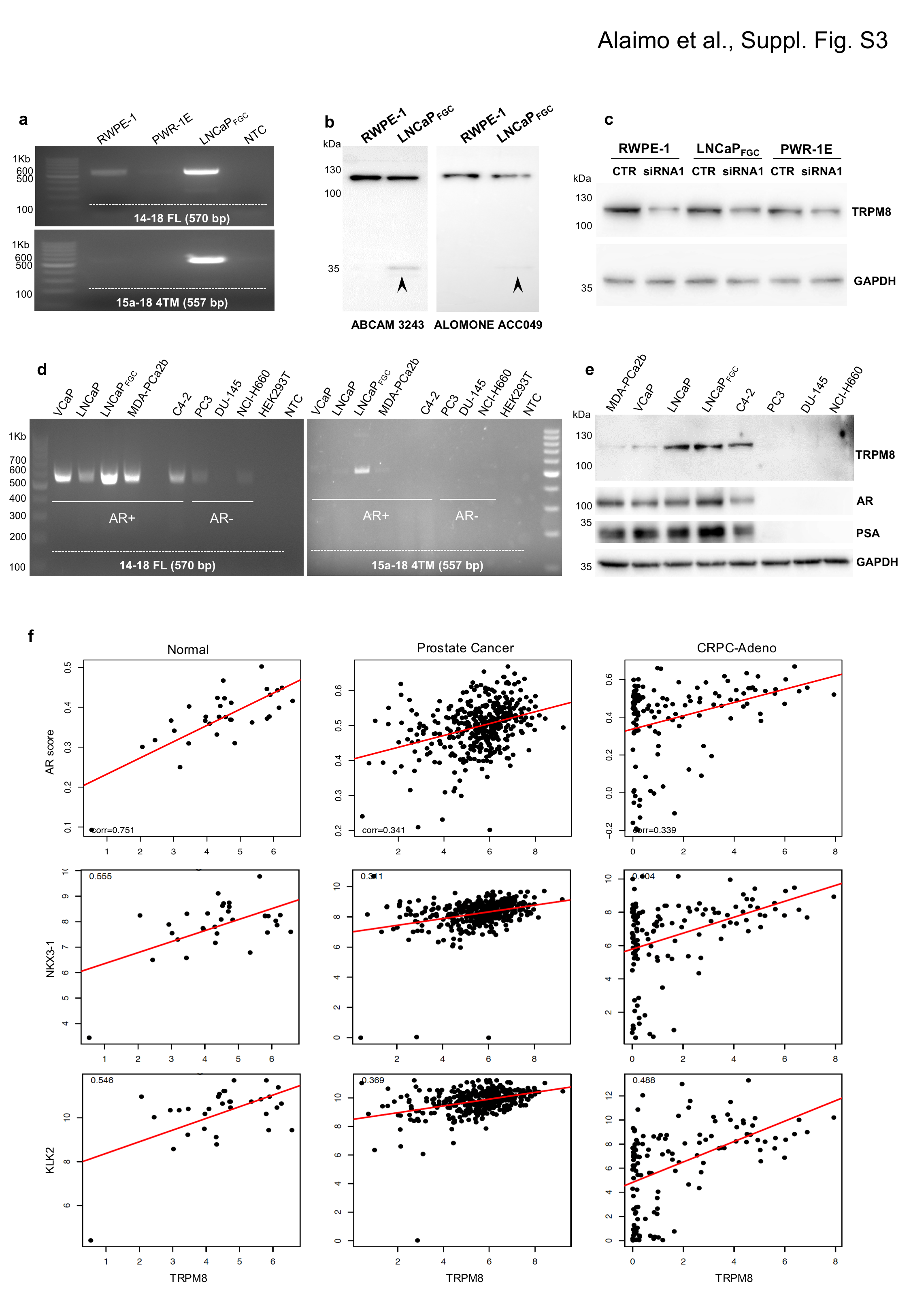

Supplement: Supplementary file 6 — Supplementary Figure S3 [file 41419_2020_3256_MOESM6_ESM.tif]

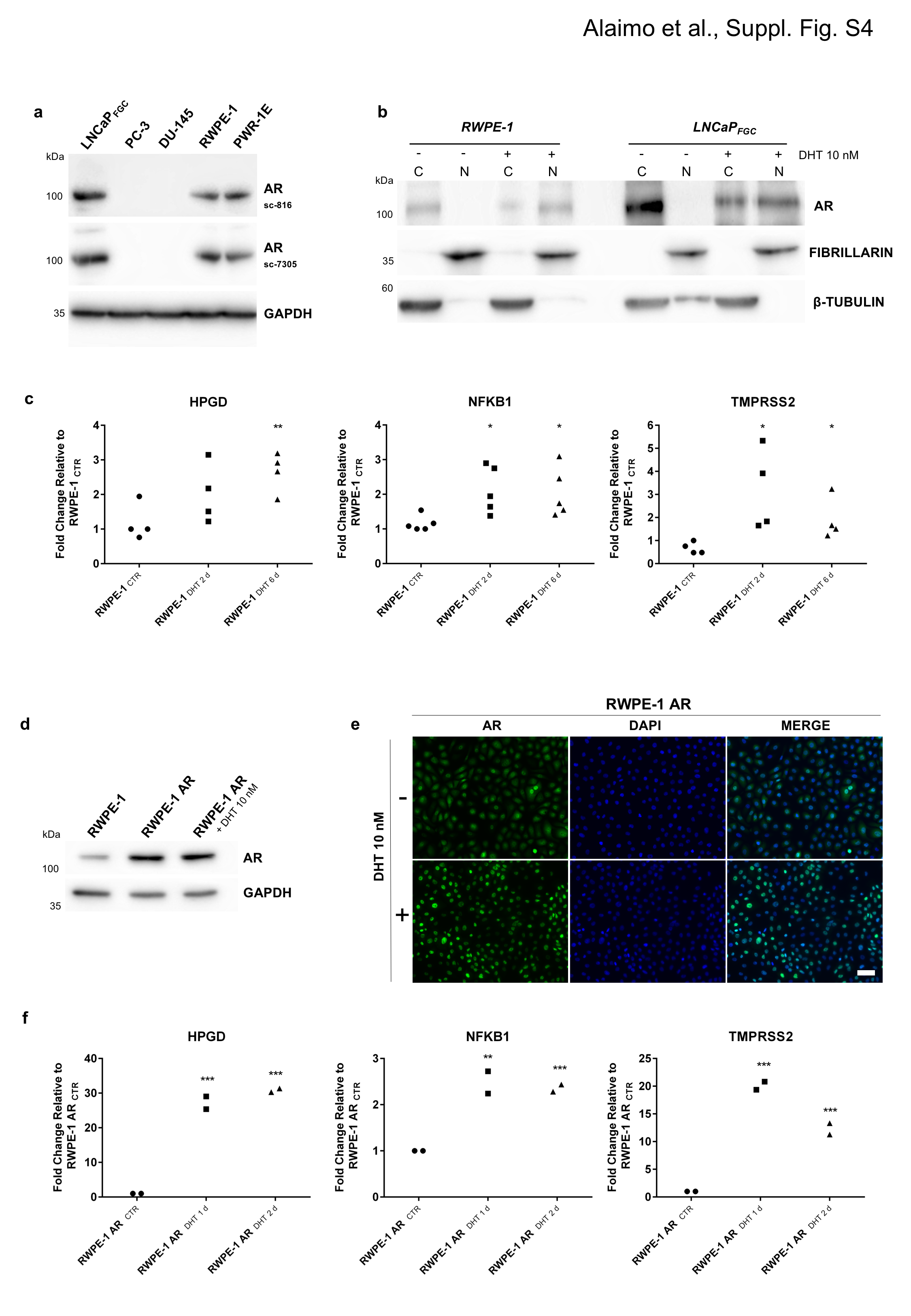

Supplement: Supplementary file 7 — Supplementary Figure S4 [file 41419_2020_3256_MOESM7_ESM.tif]

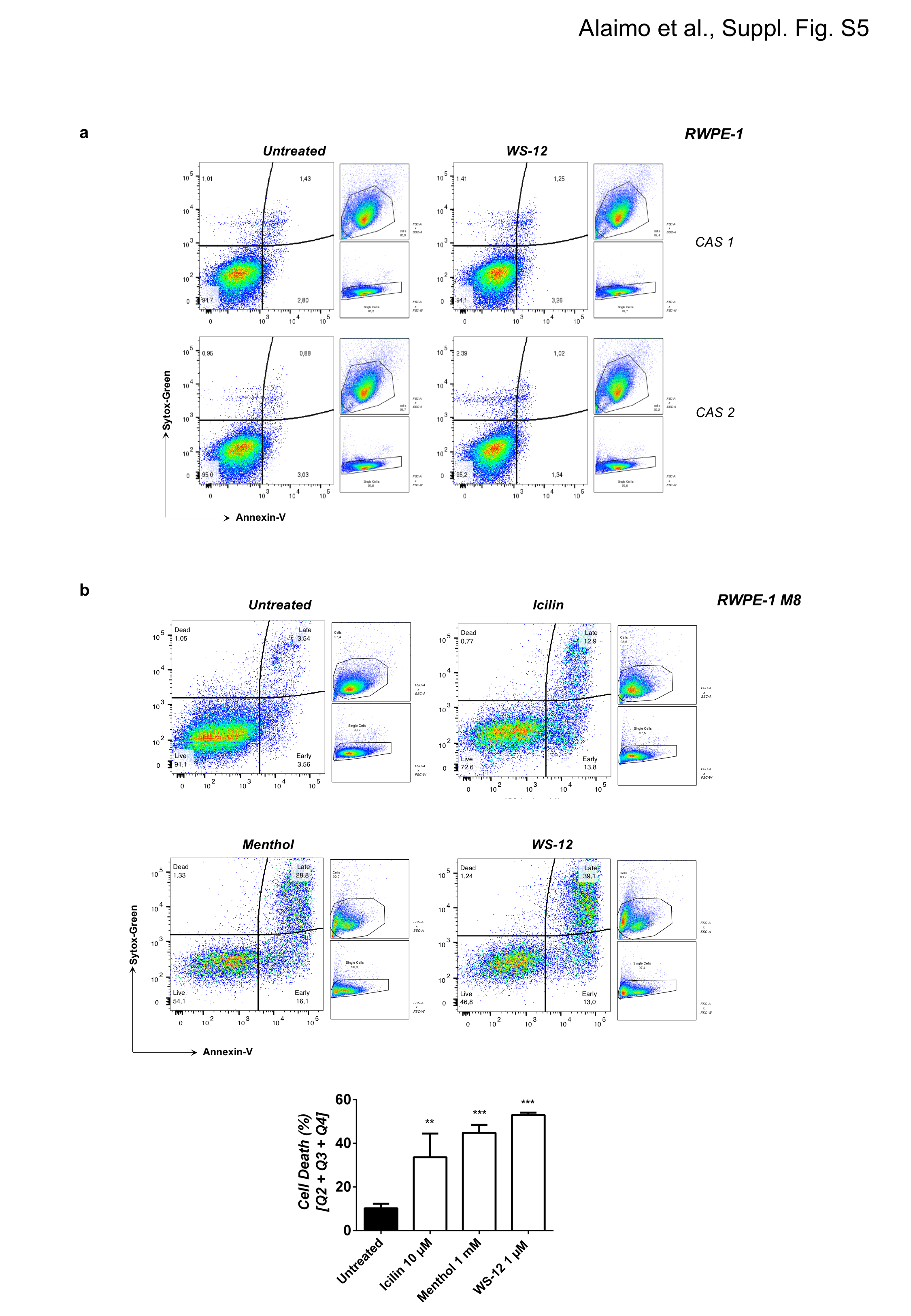

Supplement: Supplementary file 8 — Supplementary Figure S5 [file 41419_2020_3256_MOESM8_ESM.tif]

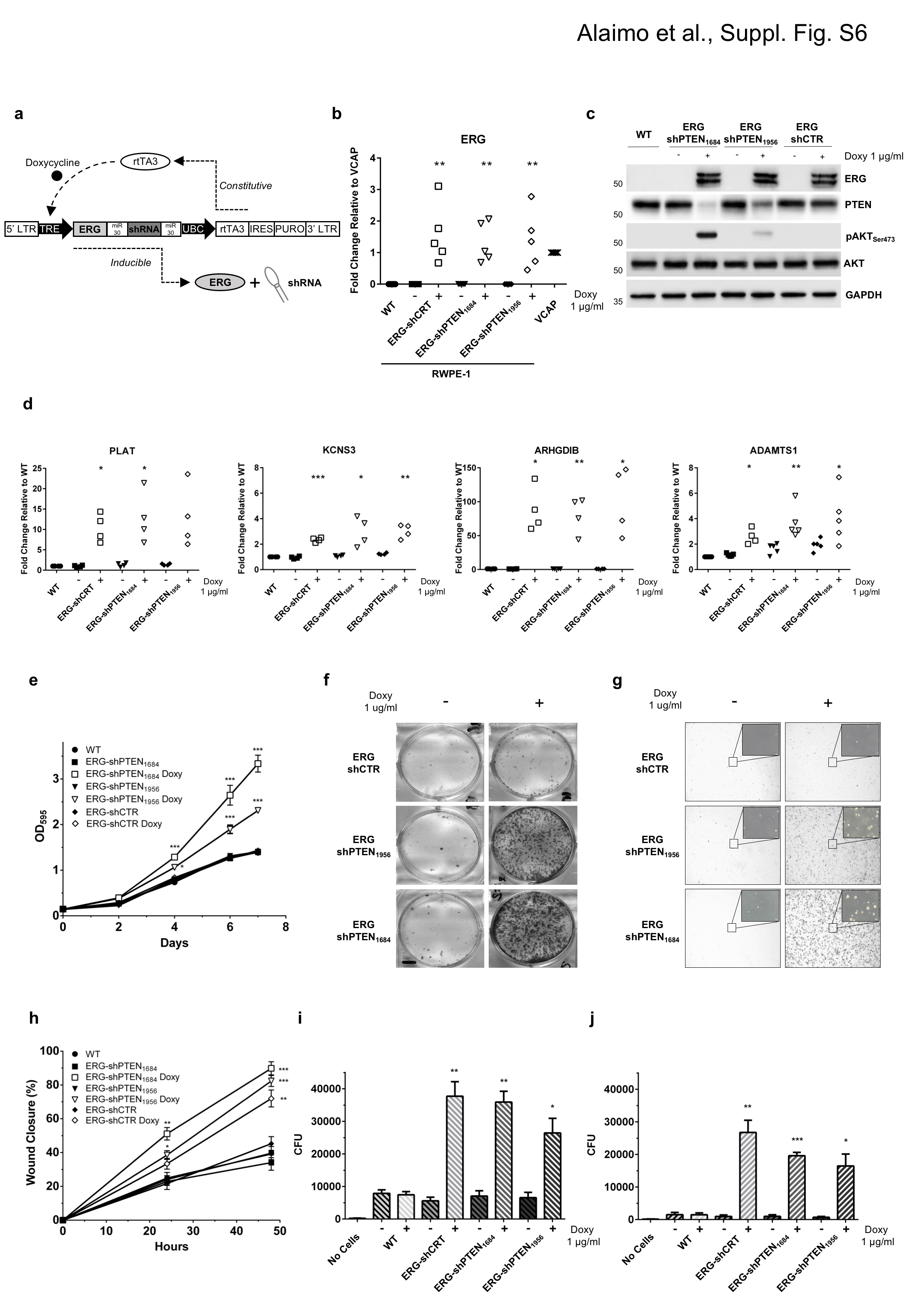

Supplement: Supplementary file 9 — Supplementary Figure S6 [file 41419_2020_3256_MOESM9_ESM.tif]

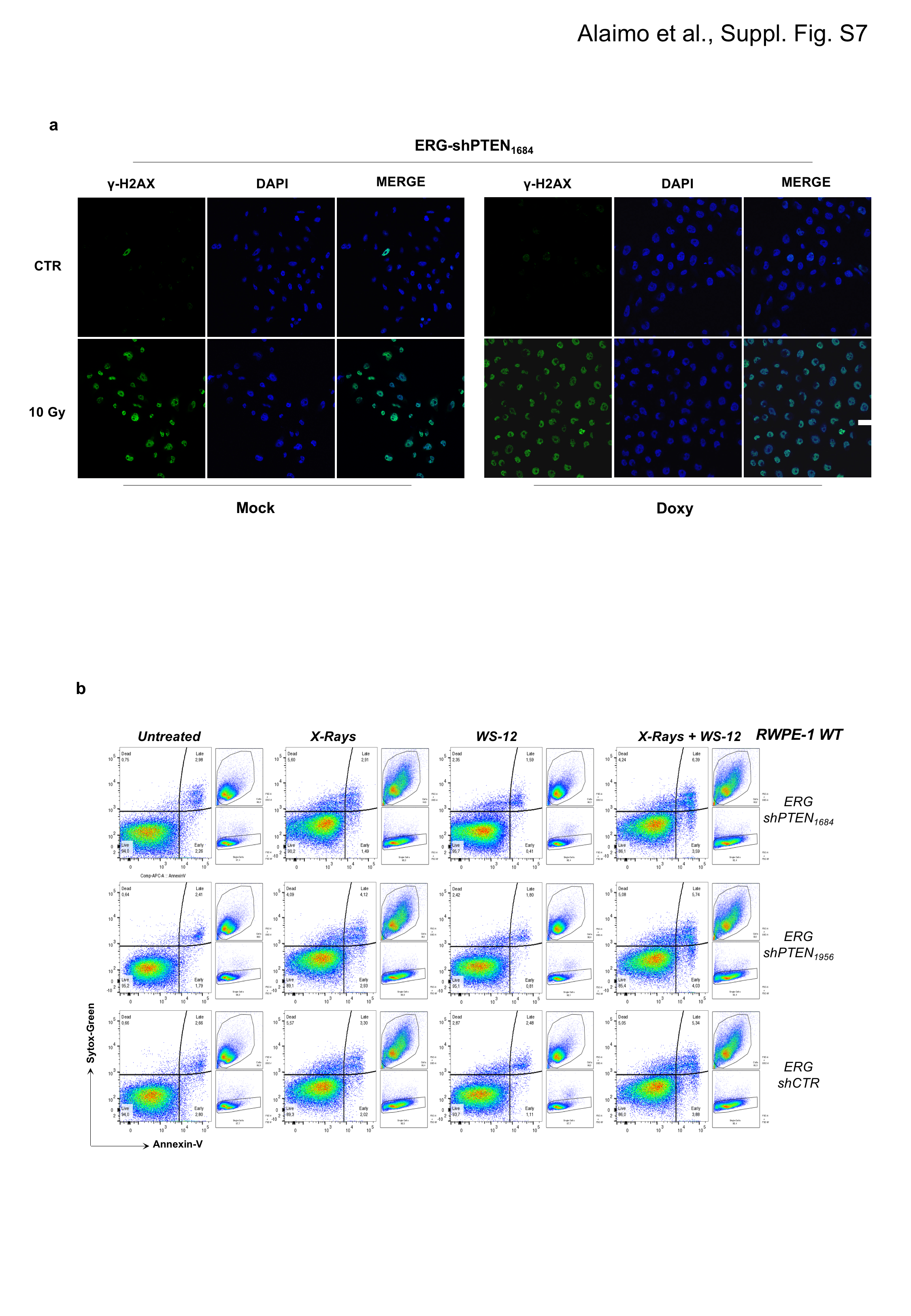

Supplement: Supplementary file 10 — Supplementary Figure S7 [file 41419_2020_3256_MOESM10_ESM.tif]

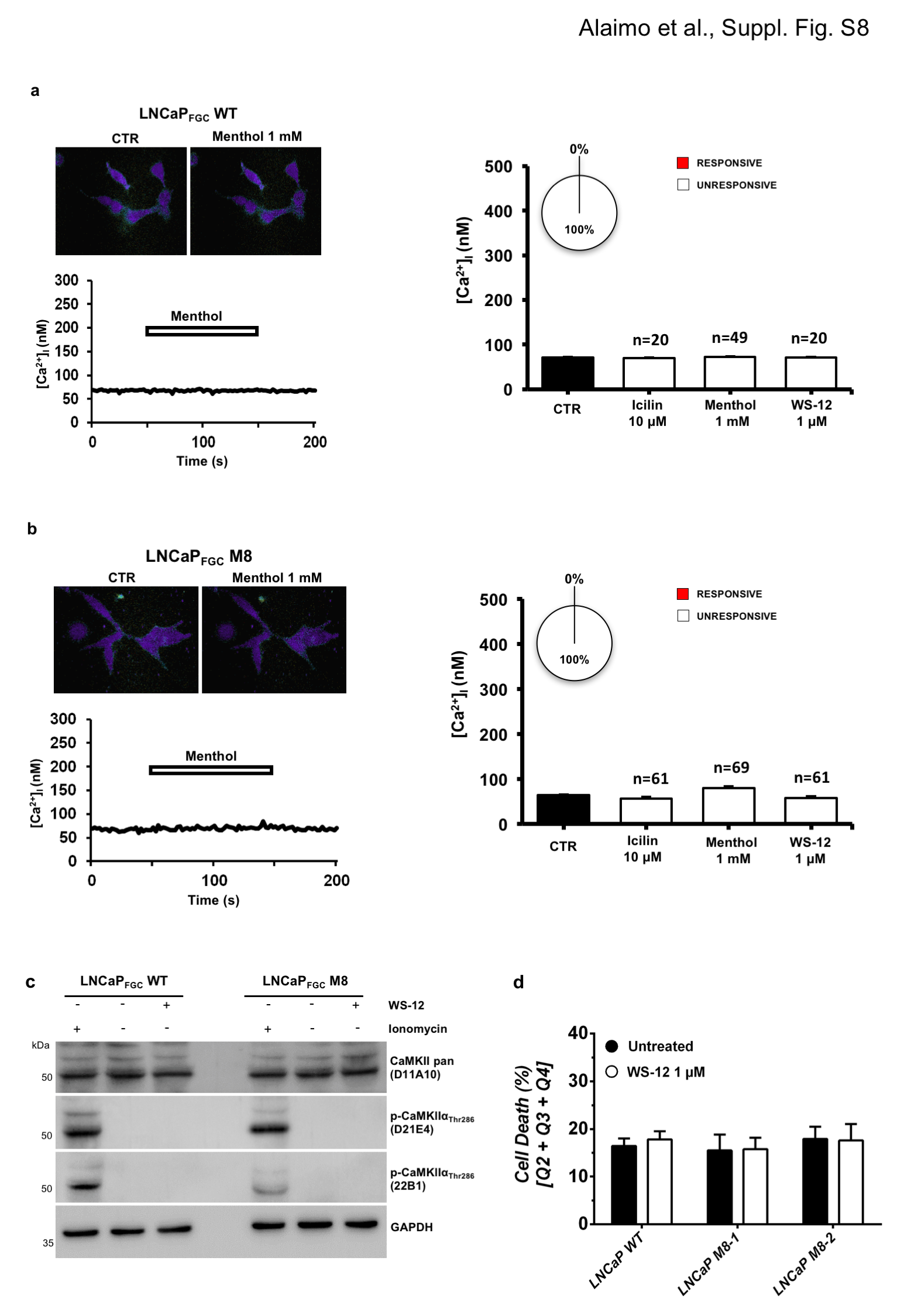

Supplement: Supplementary file 11 — Supplementary Figure S8 [file 41419_2020_3256_MOESM11_ESM.tif]

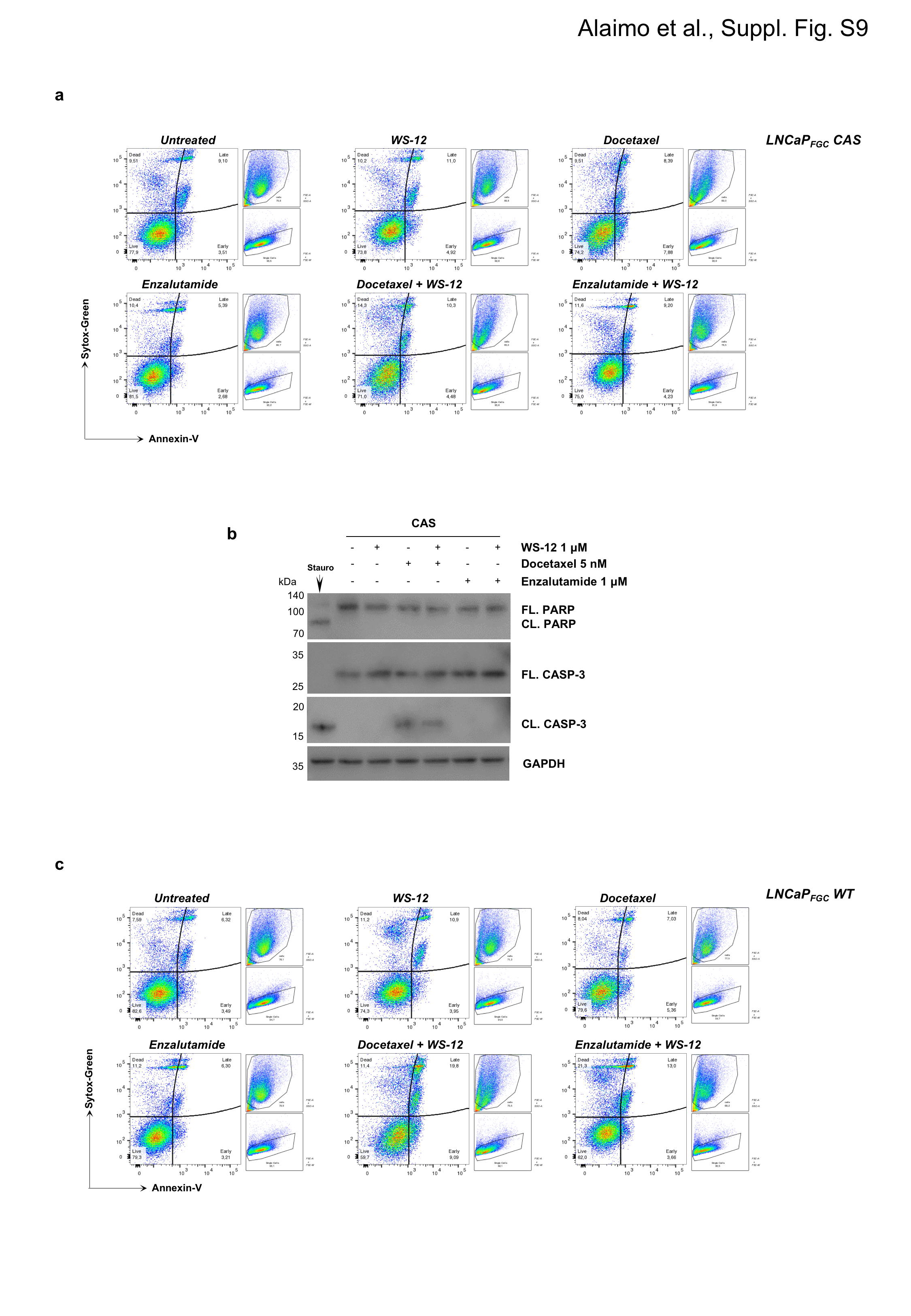

Supplement: Supplementary file 12 — Supplementary Figure S9 [file 41419_2020_3256_MOESM12_ESM.tif]
