# Supplementary material for: Calcium cytotoxicity sensitizes prostate cancer cells to standard-of-care treatments for locally advanced tumors
Source: Cell Death Dis. 2020 Dec 7;11(12):1039. doi: 10.1038/s41419-020-03256-5 (PMC7721710; doi:10.1038/s41419-020-03256-5)
Supplement: Supplementary file 13 — Supplementary Tables S1-S5 [file 41419_2020_3256_MOESM13_ESM.pdf]

Supplementary Table S1: Sequence of shRNAs

shRNA	***97-mer oligo***
shPTEN 1684h	TGCTGTTGACAGTGAGCG**ACCAGCTAAAGGTGAAGATATA**TAGTGAAGCCACAGATGTA**TATATCTTCACCTTTAGCTGGC**TGCCTACTGCCTCGGA
shPTEN 1956h	TGCTGTTGACAGTGAGCG**CGCAGATAATGACAAGGAATAT**TAGTGAAGCCACAGATGTA**ATATTCC**T**TGTCATTATCTGCA**TGCCTACTGCCTCGGA
shCRT	TGCTGTTGACAGTGAGCG**CTTACGCTGAGTACTTCGAG**TAGTGAAGCCACAGATGTA**CTCGAAGTACTCAGCGTAAG**TGCCTACTGCCTCGGAA

Sense and guide strand are highlighted in bold

Supplementary Table S2: sgRNAs and TRPM8 target sequences

sgRNA	***oligos for sgRNA cloning***	***TRPM8 target Sequence****	***TRPM8 Exon***
1	fw: caccGGTGCTTGGATTCTCACGGG<br>rev: aaacCCCGTGAGAATCCAAGCACC	gacaGGTGCTTGGATTCTCACGGGAGGcac	6
2	fw: caccgTATACGTCTGTCCTGCGACA<br>rev: aaacTGTCGCAGGACAGACGTATAc	agtaTATACGTCTGTCCTGCGACACGGacg	5
3	fw: caccgTGGCCTGATGAAGTACATCG<br>rev: aaacCGATGTACTTCATCAGGCCAc	attaTGGCCTGATGAAGTACATCGGGGagg	6
4	fw: caccGGCTAATGAGTACGAGACCC<br>rev: aaacGGGTCTCGTACTCATTAGCC	agctGGCTAATGAGTACGAGACCCGGGctg	14

*uppercase indicates sgRNA target, PAM is underlined

Supplementary Table S3: Description of primers used in this study

Human Genes	***Forward***	***Reverse***	***Reference***
ADAMTS1	ACGAGGACGAAGGGACTGAG	ATCGCTTCTTTCTTATGCTTCCA	(1)
AR	CTCTAGCCTCAATGAACTGGG	CAGGAGTACTGAATGACAGCC	
ARHGDIB	CTTACTGGAGATCTGGAAGCC	TGACAGGGTGGGAAAAGATG	
ERG	CAGCGACTATGGACAGACTTC	TGAGGTAGTGGAGATGTGAGAG	
GAPDH	AGCCACATCGCTCAGACACC	GTACTCAGCGCCAGCATCG	
HPGD	GTAAGCAAAATGGAGGTGAAGG	TGGCATTCAGTCTCACACC	
KCNS3	AGGTGAGAGTGATTTTCCAGTG	GGAGGGATTCCGATCAAAGTAG	
NFKB1	GAGTTACCTACCAGGGCTATTC	CTCTCCTCATCCTCACTCTCT	
PLAT	ACTGCTACTTTGGGAATGGG	CGGTATGTTCTGCCCAAGATC	
TBP	TTCGGAGAGTTCTGGGATTGTA	TGGACTGTTCTTCACTCTTGGC	
TMPRSS2	TCCTTCAGGTGTACTCATCTC	GCTGTCATCCACTATTCCTTG	
TRPM8	AGTCCGAGGAGCTGGCTAATG	CCAGCGTGTCCATCACATTC	F14-R18 FL
TRPM8	GTGCTGATGTCGCTGTA	CCAGCGTGTCCATCACATTC	F15a-R18 4TM
TRPM8	GATTTTCACCAATGACCGCCG	CCCCAGCAGCATTGATGTCG	F12-R15 (2)

Supplementary References

1. Carver, B.S. et al. Aberrant ERG expression cooperates with loss of PTEN to promote cancer progression in the prostate. *Nat. Genet.* **41**, 619–624 (2009).
2. Bidaux, G. et al. Prostate cell differentiation status determines transient receptor potential melastatin member 8 channel subcellular localization and function. *J. Clin. Invest.* **117**, 1647–1657 (2007)

Supplementary Table S4: List of antibodies used in this study

Primary Antibody	*Supplier*	*Catalog #*	*Type*	*WB Dilution*	*IF/IHC Dilution*
AKT	Cell Signaling	9272	Rabbit pAb	1:2000	
AR	Santa Cruz	sc-816	Rabbit pAb	1:200	1:200
AR	Santa Cruz	sc-7305	Mouse mAb	1:500	
CaMKII (pan)	Cell Signaling	4436	Rabbit mAb	1:1000	
Caspase-3	Cell Signaling	9662	Rabbit pAb	1:500	
Cleaved Caspase-3 (Asp175)	Cell Signaling	9661	Rabbit pAb	1:1000	1:200 FFPE
Cytokeratin 8	Merck Millipore	MABT329	Rat mAb		1:200 FFPE
Cytokeratin 8 + 18	Abcam	ab53280	Rabbit mAb		1:500 FFPE
Cytokeratin HMW	Leica Biosystems	PA0134	Mouse mAb		Ready to use FFTE
E-Cadherin	Abcam	ab11512	Rat mAb		1:200
ERG	Abcam	ab133264	Rabbit mAb	1:1000	
Fibrillarin	Abcam	ab4566	Mouse mAb	1:1000	
Gamma H2A.X (phospho S139)	Abcam	ab26350	Mouse mAb		1:200
GAPDH	Cell Signaling	5174	Rabbit mAb	1:4000	
Ki-67	eBioscience	BMS14-5698-82	Rat mAb		1:500 FFPE
PARP	Cell Signaling	9542	Rabbit pAb	1:1000	
Phospho-AKT (Ser473)	Cell Signaling	4060	Rabbit mAb	1:1000	
Phospho-CaMKII (Thr286)	Cell Signaling	12716	Rabbit mAb	1:1000	
Phospho-CaMKII (Thr286)	ThermoFisher Sci	MA1-047	Mouse mAb	1:1000	
Phospho-S6 Ribosomal Protein	Cell Signaling	5364	Rabbit mAb	1:1000	
PSA	Meridian Life Sci	K92110R	Rabbit pAb	1:500	
PTEN	Cell Signaling	9188	Rabbit mAb	1:2000	

S6 Ribosomal Protein	Cell Signaling	2217	Rabbit mAb	1:2000	
TRPM8	Abcam	ab3243	Rabbit pAb	1:1000	1:100<br>1:200 FFPE
TRPM8	Alomone Labs	ACC-049	Rabbit pAb	1:1000	1:100<br>1:300 FFPE
β-Actin	Sigma	A2228	Mouse mAb	1:4000	
β-Tubulin	Santa Cruz	sc-5274	Mouse mAb	1:4000	
			Secondary antibodies		
Antibody	***Fluorochrome***	***Supplier***	***Catalog #***	***Dilution***	
Donkey anti-Mouse IgG	Alexa Fluor® 488	ThermoFisher Sci	A21202	1:500	
Donkey anti-Mouse IgG	Alexa Fluor® 594	ThermoFisher Sci	A21207	1:500	
Donkey anti-Rabbit IgG	Alexa Fluor® 488	ThermoFisher Sci	A21208	1:500	
Donkey anti-Rabbit IgG	Alexa Fluor® 594	ThermoFisher Sci	A21203	1:500	
Donkey anti-Rat IgG	Alexa Fluor® 488	ThermoFisher Sci	A11055	1:500	
Donkey anti-Rat IgG	Alexa Fluor® 594	ThermoFisher Sci	A21209	1:500	
Horse anti-Mouse IgG HRP	None	Cell Signaling	7076	1:1000 – 1:8000	
Goat anti-Rabbit IgG HRP	None	Cell Signaling	7074	1:1000 – 1:8000	

FFPE: Formalin Fixed Paraffin Embedded sections

Supplementary Table S5: Prostate cancer tissue microarray

Description	Prostate cancer tissue array, with pathology grade, Gleason grade, Gleason score, TNM and clinical stage, 64 cases/192 cores											
Position	No.	Age	Sex	Organ	Pathology diagnosis	TNM	Grade	Stage	Type	Tissue ID.	Gleason Score	Gleason Grade
A1	1	68	M	Prostate	Adenocarcinoma	T2N0M0	1	I	Malignant	Mpr130039	2+3	3
A2	2	68	M	Prostate	Adenocarcinoma	T2N0M0	1	I	Malignant	Mpr130039	2+3	3
A3	3	68	M	Prostate	Adenocarcinoma	T2N0M0	1	I	Malignant	Mpr130039	2+3	3
A4	4	63	M	Prostate	Adenocarcinoma	T3N0M0	1	III	Malignant	Mpr130009	1+2	2
A5	5	63	M	Prostate	Adenocarcinoma	T3N0M0	1	III	Malignant	Mpr130009	1+2	2
A6	6	63	M	Prostate	Adenocarcinoma	T3N0M0	1	III	Malignant	Mpr130009	1+2	2
A7	7	74	M	Prostate	Adenocarcinoma	T4N1M0	1	IV	Malignant	Mpr040041	1+2	2
A8	8	74	M	Prostate	Adenocarcinoma	T4N1M0	1	IV	Malignant	Mpr040041	1+2	2
A9	9	74	M	Prostate	Adenocarcinoma	T4N1M0	1	IV	Malignant	Mpr040041	1+2	2
A10	10	26	M	Prostate	Adenocarcinoma	T2N0M0	-	I	Malignant	Mpr060006	-	-
A11	11	26	M	Prostate	Adenocarcinoma	T2N0M0	-	I	Malignant	Mpr060006	-	-
A12	12	26	M	Prostate	Adenocarcinoma	T2N0M0	-	I	Malignant	Mpr060006	-	
A13	13	66	M	Prostate	Adenocarcinoma	T2N0M0	1	I	Malignant	Mpr130012	2+2	2
A14	14	66	M	Prostate	Adenocarcinoma	T2N0M0	1	I	Malignant	Mpr130012	2+2	2
A15	15	66	M	Prostate	Adenocarcinoma	T2N0M0	1	I	Malignant	Mpr130012	2+2	2
A16	16	66	M	Prostate	Adenocarcinoma	T3N1M1	1	IV	Malignant	Mpr020491	1+2	2
B1	17	66	M	Prostate	Adenocarcinoma	T3N1M1	1	IV	Malignant	Mpr020491	1+2	2
B2	18	66	M	Prostate	Adenocarcinoma	T3N1M1	1	IV	Malignant	Mpr020491	1+2	2
B3	19	57	M	Prostate	Adenocarcinoma	T2N0M0	1	I	Malignant	Mpr130032	2+3	3
B4	20	57	M	Prostate	Adenocarcinoma	T2N0M0	1	I	Malignant	Mpr130032	2+3	3
B5	21	57	M	Prostate	Adenocarcinoma	T2N0M0	1	I	Malignant	Mpr130032	2+3	3
B6	22	71	M	Prostate	Adenocarcinoma	T2N0M0	1	I	Malignant	Mpr080049	2+2	2
B7	23	71	M	Prostate	Adenocarcinoma	T2N0M0	-	I	Malignant	Mpr080049	-	-
B8	24	71	M	Prostate	Adenocarcinoma	T2N0M0	1	I	Malignant	Mpr080049	2+2	2
B9	25	75	M	Prostate	Adenocarcinoma	T2N0M0	1	I	Malignant	Mpr030402	2+2	2
B10	26	75	M	Prostate	Adenocarcinoma	T2N0M0	1	I	Malignant	Mpr030402	2+2	2
B11	27	75	M	Prostate	Adenocarcinoma	T2N0M0	1	I	Malignant	Mpr030402	2+2	2
B12	28	72	M	Prostate	Adenocarcinoma	T2N0M0	2	IIA	Malignant	Mpr040048	3+4	4
B13	29	72	M	Prostate	Adenocarcinoma	T2N0M0	2	IIA	Malignant	Mpr040048	3+4	4
B14	30	72	M	Prostate	Adenocarcinoma	T2N0M0	2	IIA	Malignant	Mpr040048	3+4	4
B15	31	73	M	Prostate	Adenocarcinoma	T2N0M0	2	I	Malignant	Mpr130014	3+3	3
B16	32	73	M	Prostate	Adenocarcinoma	T2N0M0	2	I	Malignant	Mpr130014	3+3	3
C1	33	73	M	Prostate	Adenocarcinoma	T2N0M0	2	I	Malignant	Mpr130014	3+3	3
C2	34	67	M	Prostate	Adenocarcinoma	T2N0M0	2	I	Malignant	Mpr030220	3+3	3
C3	35	67	M	Prostate	Adenocarcinoma	T2N0M0	2	I	Malignant	Mpr030220	3+3	3
C4	36	67	M	Prostate	Adenocarcinoma	T2N0M0	2	I	Malignant	Mpr030220	3+3	3
C5	37	65	M	Prostate	Adenocarcinoma	T2N0M0	1	I	Malignant	Mpr030031	2+3	3
C6	38	65	M	Prostate	Adenocarcinoma	T2N0M0	1	I	Malignant	Mpr030031	2+3	3
C7	39	65	M	Prostate	Adenocarcinoma	T2N0M0	1	I	Malignant	Mpr030031	2+3	3
C8	40	65	M	Prostate	Adenocarcinoma	T2N0M0	2	I	Malignant	Mpr070087	3+3	3
C9	41	65	M	Prostate	Adenocarcinoma	T2N0M0	2	I	Malignant	Mpr070087	3+3	3
C10	42	65	M	Prostate	Adenocarcinoma	T2N0M0	2	I	Malignant	Mpr070087	3+3	3
C11	43	83	M	Prostate	Adenocarcinoma	T4N1M1	-	IV	Malignant	Mpr020524	-	-
C12	44	83	M	Prostate	Adenocarcinoma	T4N1M1	-	IV	Malignant	Mpr020524	-	-
C13	45	83	M	Prostate	Adenocarcinoma	T4N1M1	-	IV	Malignant	Mpr020524	-	-
C14	46	70	M	Prostate	Adenocarcinoma	T2N1M1	2	IV	Malignant	Mpr020345	3+4	4
C15	47	70	M	Prostate	Adenocarcinoma	T2N1M1	2	IV	Malignant	Mpr020345	3+4	4

C16	48	70	M	Prostate	Adenocarcinoma	T2N1M1	2	IV	Malignant	Mpr020345	3+4	4
D1	49	56	M	Prostate	Adenocarcinoma	T2N0M0	2	IIA	Malignant	Mpr040206	3+4	4
D2	50	56	M	Prostate	Adenocarcinoma	T2N0M0	2	IIA	Malignant	Mpr040206	3+4	4
D3	51	56	M	Prostate	Adenocarcinoma	T2N0M0	2	IIA	Malignant	Mpr040206	3+4	4
D4	52	75	M	Prostate	Adenocarcinoma	T2N0M0	2	I	Malignant	Mpr030207	2+4	4
D5	53	75	M	Prostate	Adenocarcinoma	T2N0M0	2	I	Malignant	Mpr030207	2+4	4
D6	54	75	M	Prostate	Adenocarcinoma	T2N0M0	2	I	Malignant	Mpr030207	2+4	4
D7	55	64	M	Prostate	Adenocarcinoma	T2N0M0	2	I	Malignant	Mpr010054	3+3	3
D8	56	64	M	Prostate	Adenocarcinoma	T2N0M0	2	I	Malignant	Mpr010054	3+3	3
D9	57	64	M	Prostate	Adenocarcinoma	T2N0M0	2	I	Malignant	Mpr010054	3+3	3
D10	58	71	M	Prostate	Adenocarcinoma	T2N0M0	2	IIA	Malignant	Mpr130007	3+4	4
D11	59	71	M	Prostate	Adenocarcinoma	T2N0M0	2	IIA	Malignant	Mpr130007	3+4	4
D12	60	71	M	Prostate	Adenocarcinoma	T2N0M0	2	IIA	Malignant	Mpr130007	3+4	4
D13	61	82	M	Prostate	Adenocarcinoma	T2N0M0	2	IIA	Malignant	Mpr100019	3+4	4
D14	62	82	M	Prostate	Adenocarcinoma	T2N0M0	2	IIA	Malignant	Mpr100019	3+4	4
D15	63	82	M	Prostate	Adenocarcinoma	T2N0M0	2	IIA	Malignant	Mpr100019	3+4	4
D16	64	73	M	Prostate	Adenocarcinoma	T3N0M0	2	III	Malignant	Mpr090005	3+3	3
E1	65	73	M	Prostate	Adenocarcinoma	T3N0M0	2	III	Malignant	Mpr090005	3+3	3
E2	66	73	M	Prostate	Adenocarcinoma	T3N0M0	2	III	Malignant	Mpr090005	3+3	3
E3	67	60	M	Prostate	Adenocarcinoma	T3N1M0	2	IV	Malignant	Mpr080011	3+4	4
E4	68	60	M	Prostate	Adenocarcinoma	T3N1M0	2	IV	Malignant	Mpr080011	3+4	4
E5	69	60	M	Prostate	Adenocarcinoma	T3N1M0	2	IV	Malignant	Mpr080011	3+4	4
E6	70	64	M	Prostate	Adenocarcinoma	T2N0M0	2	IIA	Malignant	Mpr070118	3+4	4
E7	71	64	M	Prostate	Adenocarcinoma	T2N0M0	2	IIA	Malignant	Mpr070118	3+4	4
E8	72	64	M	Prostate	Adenocarcinoma	T2N0M0	2	IIA	Malignant	Mpr070118	3+4	4
E9	73	69	M	Prostate	Adenocarcinoma	T2N0M0	2	I	Malignant	Mpr020114	3+3	3
E10	74	69	M	Prostate	Adenocarcinoma	T2N0M0	2	I	Malignant	Mpr020114	3+3	3
E11	75	69	M	Prostate	Adenocarcinoma	T2N0M0	2	I	Malignant	Mpr020114	3+3	3
E12	76	62	M	Prostate	Adenocarcinoma	T2N0M0	2	IIA	Malignant	Mpr050083	3+4	4
E13	77	62	M	Prostate	Adenocarcinoma	T2N0M0	2	IIA	Malignant	Mpr050083	3+4	4
E14	78	62	M	Prostate	Adenocarcinoma	T2N0M0	2	IIA	Malignant	Mpr050083	3+4	4
E15	79	73	M	Prostate	Adenocarcinoma	T2N0M0	2	IIA	Malignant	Mpr050122	3+4	4
E16	80	73	M	Prostate	Adenocarcinoma	T2N0M0	2	IIA	Malignant	Mpr050122	3+4	4
F1	81	73	M	Prostate	Adenocarcinoma	T2N0M0	2	IIA	Malignant	Mpr050122	3+4	4
F2	82	61	M	Prostate	Adenocarcinoma	T2N0M0	2	IIA	Malignant	Mpr070019	3+4	4
F3	83	61	M	Prostate	Adenocarcinoma	T2N0M0	2	IIA	Malignant	Mpr070019	3+4	4
F4	84	61	M	Prostate	Adenocarcinoma	T2N0M0	2	IIA	Malignant	Mpr070019	3+4	4
F5	85	70	M	Prostate	Adenocarcinoma	T3N0M0	2	III	Malignant	Mpr060100	3+4	4
F6	86	70	M	Prostate	Adenocarcinoma	T3N0M0	2	III	Malignant	Mpr060100	3+4	4
F7	87	70	M	Prostate	Adenocarcinoma	T3N0M0	2	III	Malignant	Mpr060100	3+4	4
F8	88	73	M	Prostate	Adenocarcinoma	T2N0M0	2	IIA	Malignant	Mpr020053	3+4	4
F9	89	73	M	Prostate	Adenocarcinoma	T2N0M0	2	IIA	Malignant	Mpr020053	3+4	4
F10	90	73	M	Prostate	Adenocarcinoma	T2N0M0	2	IIA	Malignant	Mpr020053	3+4	4
F11	91	76	M	Prostate	Adenocarcinoma	T2N0M0	2	I	Malignant	Mpr040106	3+3	3
F12	92	76	M	Prostate	Adenocarcinoma	T2N0M0	2	I	Malignant	Mpr040106	3+3	3
F13	93	76	M	Prostate	Adenocarcinoma	T2N0M0	2	I	Malignant	Mpr040106	3+3	3
F14	94	69	M	Prostate	Adenocarcinoma	T3N0M0	2	III	Malignant	Mpr030166	3+3	3
F15	95	69	M	Prostate	Adenocarcinoma (sparse)	T3N0M0	-	III	Malignant	Mpr030166	-	-
F16	96	69	M	Prostate	Adenocarcinoma	T3N0M0	2	III	Malignant	Mpr030166	3+3	3
G1	97	65	M	Prostate	Adenocarcinoma	T2N0M0	3	IIB	Malignant	Mpr040034	3+5	5
G2	98	65	M	Prostate	Adenocarcinoma	T2N0M0	3	IIB	Malignant	Mpr040034	3+5	5

G3	99	65	M	Prostate	Adenocarcinoma	T2N0M0	3	IIB	Malignant	Mpr040034	3+5	5
G4	100	-	M	Prostate	Adenocarcinoma	T2N0M0	-	IIB	Malignant	Mtt110008	-	-
G5	101	-	M	Prostate	Adenocarcinoma	T2N0M0	-	IIB	Malignant	Mtt110008	-	-
G6	102	-	M	Prostate	Adenocarcinoma	T2N0M0	-	IIB	Malignant	Mtt110008	-	-
G7	103	62	M	Prostate	Adenocarcinoma	T3N0M0	2	III	Malignant	Mpr040089	3+4	4
G8	104	62	M	Prostate	Adenocarcinoma	T3N0M0	2	III	Malignant	Mpr040089	3+4	4
G9	105	62	M	Prostate	Adenocarcinoma	T3N0M0	2	III	Malignant	Mpr040089	3+4	4
G10	106	76	M	Prostate	Adenocarcinoma	T3N0M0	3	III	Malignant	Mpr020387	4+5	5
G11	107	76	M	Prostate	Adenocarcinoma	T3N0M0	3	III	Malignant	Mpr020387	4+5	5
G12	108	76	M	Prostate	Adenocarcinoma	T3N0M0	3	III	Malignant	Mpr020387	4+5	5
G13	109	82	M	Prostate	Adenocarcinoma	T3N0M0	2	III	Malignant	Mpr040075	3+4	4
G14	110	82	M	Prostate	Adenocarcinoma	T3N0M0	2	III	Malignant	Mpr040075	3+4	4
G15	111	82	M	Prostate	Adenocarcinoma	T3N0M0	2	III	Malignant	Mpr040075	3+4	4
G16	112	64	M	Prostate	Adenocarcinoma	T3N0M0	3	III	Malignant	Mpr020003	4+5	5
H1	113	64	M	Prostate	Adenocarcinoma	T3N0M0	3	III	Malignant	Mpr020003	4+5	5
H2	114	64	M	Prostate	Adenocarcinoma	T3N0M0	3	III	Malignant	Mpr020003	4+5	5
H3	115	64	M	Prostate	Adenocarcinoma	T3N0M0	2	III	Malignant	Mpr030359	3+3	3
H4	116	64	M	Prostate	Adenocarcinoma	T3N0M0	2	III	Malignant	Mpr030359	3+3	3
H5	117	64	M	Prostate	Adenocarcinoma	T3N0M0	2	III	Malignant	Mpr030359	3+3	3
H6	118	78	M	Prostate	Adenocarcinoma	T4N0M0	3	IV	Malignant	Mpr030228	4+5	5
H7	119	78	M	Prostate	Adenocarcinoma	T4N0M0	3	IV	Malignant	Mpr030228	4+5	5
H8	120	78	M	Prostate	Adenocarcinoma	T4N0M0	3	IV	Malignant	Mpr030228	4+5	5
H9	121	64	M	Prostate	Adenocarcinoma	T2N0M0	3	IIB	Malignant	Mpr080013	3+5	5
H10	122	64	M	Prostate	Adenocarcinoma	T2N0M0	3	IIB	Malignant	Mpr080013	3+5	5
H11	123	64	M	Prostate	Adenocarcinoma	T2N0M0	3	IIB	Malignant	Mpr080013	3+5	5
H12	124	66	M	Prostate	Adenocarcinoma	T3N0M0	3	III	Malignant	Mpr060047	3+5	5
H13	125	66	M	Prostate	Adenocarcinoma	T3N0M0	3	III	Malignant	Mpr060047	3+5	5
H14	126	66	M	Prostate	Adenocarcinoma	T3N0M0	3	III	Malignant	Mpr060047	3+5	5
H15	127	64	M	Prostate	Adenocarcinoma	T2N0M0	2	IIA	Malignant	Mpr040171	3+4	4
H16	128	64	M	Prostate	Adenocarcinoma	T2N0M0	2	IIA	Malignant	Mpr040171	3+4	4
I1	129	64	M	Prostate	Adenocarcinoma	T2N0M0	2	IIA	Malignant	Mpr040171	3+4	4
I2	130	73	M	Prostate	Adenocarcinoma	T4N0M0	-	IV	Malignant	Mpr030188	-	-
I3	131	73	M	Prostate	Adenocarcinoma	T4N0M0	3	IV	Malignant	Mpr030188	5+5	5
I4	132	73	M	Prostate	Adenocarcinoma	T4N0M0	3	IV	Malignant	Mpr030188	5+5	5
I5	133	63	M	Prostate	Adenocarcinoma	T2N0M0	3	IIB	Malignant	Mpr030347	4+5	5
I6	134	63	M	Prostate	Adenocarcinoma	T2N0M0	3	IIB	Malignant	Mpr030347	4+5	5
I7	135	63	M	Prostate	Adenocarcinoma	T2N0M0	3	IIB	Malignant	Mpr030347	4+5	5
I8	136	60	M	Prostate	Adenocarcinoma	T3N1M0	3	IV	Malignant	Mpr030236	5+5	5
I9	137	60	M	Prostate	Adenocarcinoma	T3N1M0	3	IV	Malignant	Mpr030236	5+5	5
I10	138	60	M	Prostate	Adenocarcinoma	T3N1M0	3	IV	Malignant	Mpr030236	5+5	5
I11	139	80	M	Prostate	Adenocarcinoma	T3N0M0	3	III	Malignant	Mpr020382	4+5	5
I12	140	80	M	Prostate	Adenocarcinoma	T3N0M0	3	III	Malignant	Mpr020382	4+5	5
I13	141	80	M	Prostate	Adenocarcinoma	T3N0M0	3	III	Malignant	Mpr020382	4+5	5
I14	142	73	M	Prostate	Adenocarcinoma	T2N0M0	3	IIB	Malignant	Mpr130034	5+4	5
I15	143	73	M	Prostate	Adenocarcinoma	T2N0M0	3	IIB	Malignant	Mpr130034	5+4	5
I16	144	73	M	Prostate	Adenocarcinoma	T2N0M0	3	IIB	Malignant	Mpr130034	5+4	5
J1	145	64	M	Prostate	Adenocarcinoma	T2N0M0	2	IIA	Malignant	Mpr130033	3+4	3
J2	146	64	M	Prostate	Adenocarcinoma	T2N0M0	-	IIA	Malignant	Mpr130033	-	-
J3	147	64	M	Prostate	Adenocarcinoma	T2N0M0	2	IIA	Malignant	Mpr130033	3+4	4
J4	148	68	M	Prostate	Adenocarcinoma	T2N0M0	-	IIA	Malignant	Mpr130011	-	-
J5	149	68	M	Prostate	Adenocarcinoma	T2N0M0	-	IIA	Malignant	Mpr130011	-	-

J6	150	68	M	Prostate	Adenocarcinoma	T2N0M0	3	IIB	Malignant	Mpr130011	5+5	5
J7	151	87	M	Prostate	Adenocarcinoma	T2N0M0	3	IIB	Malignant	Mpr020242	5+4	5
J8	152	87	M	Prostate	Adenocarcinoma	T2N0M0	3	IIB	Malignant	Mpr020242	5+4	5
J9	153	87	M	Prostate	Adenocarcinoma	T2N0M0	3	IIB	Malignant	Mpr020242	5+4	5
J10	154	72	M	Prostate	Adenocarcinoma	T3N0M0	3	III	Malignant	Mpr010051	5+4	5
J11	155	72	M	Prostate	Adenocarcinoma	T3N0M0	-	III	Malignant	Mpr010051	-	-
J12	156	72	M	Prostate	Adenocarcinoma	T3N0M0	-	III	Malignant	Mpr010051	-	-
J13	157	82	M	Prostate	Adenocarcinoma	T2N0M0	3	IIB	Malignant	Mpr030147	5+4	5
J14	158	82	M	Prostate	Adenocarcinoma	T2N0M0	3	IIB	Malignant	Mpr030147	5+4	5
J15	159	82	M	Prostate	Adenocarcinoma	T2N0M0	3	IIB	Malignant	Mpr030147	5+4	5
J16	160	62	M	Prostate	Adenocarcinoma	T2N0M0	3	IIB	Malignant	Mpr040163	4+5	5
K1	161	62	M	Prostate	Adenocarcinoma	T2N0M0	3	IIB	Malignant	Mpr040163	4+5	5
K2	162	62	M	Prostate	Adenocarcinoma	T2N0M0	3	IIB	Malignant	Mpr040163	4+5	5
K3	163	77	M	Prostate	Adenocarcinoma	T2N0M0	3	IIB	Malignant	Mpr040215	5+5	5
K4	164	77	M	Prostate	Adenocarcinoma	T2N0M0	3	IIB	Malignant	Mpr040215	5+5	5
K5	165	77	M	Prostate	Adenocarcinoma	T2N0M0	3	IIB	Malignant	Mpr040215	5+5	5
K6	166	60	M	Prostate	Adenocarcinoma	T2N0M0	3	IIB	Malignant	Mpr020323	5+5	5
K7	167	60	M	Prostate	Adenocarcinoma	T2N0M0	3	IIB	Malignant	Mpr020323	5+5	5
K8	168	60	M	Prostate	Adenocarcinoma	T2N0M0	3	IIB	Malignant	Mpr020323	5+5	5
K9	169	81	M	Prostate	Adenocarcinoma	T3N0M0	3	III	Malignant	Mpr020317	5+4	5
K10	170	81	M	Prostate	Adenocarcinoma	T3N0M0	3	III	Malignant	Mpr020317	5+4	5
K11	171	81	M	Prostate	Adenocarcinoma	T3N0M0	3	III	Malignant	Mpr020317	5+4	5
K12	172	70	M	Prostate	Small acinar cancer	T2N0M0	-	IIA	Malignant	Mpr020038	-	-
K13	173	70	M	Prostate	Small acinar cancer	T2N0M0	-	IIA	Malignant	Mpr020038	-	-
K14	174	70	M	Prostate	Small acinar cancer	T2N0M0	-	IIA	Malignant	Mpr020038	-	-
K15	175	45	M	Prostate	Prostate tissue	-	-	-	Normal	Mpr07N030	-	-
K16	176	45	M	Prostate	Prostate tissue	-	-	-	Normal	Mpr07N030	-	-
L1	177	45	M	Prostate	Prostate tissue	-	-	-	Normal	Mpr07N030	-	-
L2	178	40	M	Prostate	Prostate tissue	-	-	-	Normal	Mpr08N003	-	-
L3	179	40	M	Prostate	Prostate tissue	-	-	-	Normal	Mpr08N003	-	-
L4	180	40	M	Prostate	Prostate tissue	-	-	-	Normal	Mpr08N003	-	-
L5	181	30	M	Prostate	Prostate tissue	-	-	-	Normal	Mpr08N004	-	-
L6	182	30	M	Prostate	Prostate tissue	-	-	-	Normal	Mpr08N004	-	-
L7	183	30	M	Prostate	Prostate tissue	-	-	-	Normal	Mpr08N004	-	-
L8	184	32	M	Prostate	Prostate tissue	-	-	-	Normal	Mpr08N007	-	-
L9	185	32	M	Prostate	Prostate tissue	-	-	-	Normal	Mpr08N007	-	-
L10	186	32	M	Prostate	Prostate tissue	-	-	-	Normal	Mpr08N007	-	-
L11	187	30	M	Prostate	Prostate tissue	-	-	-	Normal	Mpr08N014	-	-
L12	188	30	M	Prostate	Prostate tissue	-	-	-	Normal	Mpr08N014	-	-
L13	189	30	M	Prostate	Prostate tissue	-	-	-	Normal	Mpr08N014	-	-
L14	190	35	M	Prostate	Prostate tissue	-	-	-	Normal	Mpr08N011	-	-
L15	191	35	M	Prostate	Prostate tissue	-	-	-	Normal	Mpr08N011	-	-
L16	192	35	M	Prostate	Prostate tissue	-	-	-	Normal	Mpr08N011	-	-
